# mNG-tagged fusion proteins and nanobodies to visualize tropomyosins in yeast and mammalian cells

**DOI:** 10.1242/jcs.260288

**Published:** 2022-09-23

**Authors:** Tomoyuki Hatano, Tzer Chyn Lim, Ingrid Billault-Chaumartin, Anubhav Dhar, Ying Gu, Teresa Massam-Wu, William Scott, Sushmitha Adishesha, Bernardo Chapa-y-Lazo, Luke Springall, Lavanya Sivashanmugam, Masanori Mishima, Sophie G. Martin, Snezhana Oliferenko, Saravanan Palani, Mohan K. Balasubramanian

**Affiliations:** ^1^ Centre for Mechanochemical Cell Biology and Division of Biomedical Sciences, Warwick Medical School, Warwick CV4 7AL, UK; ^2^ Department of Fundamental Microbiology, Faculty of Biology and Medicine, University of Lausanne, Biophore Building, CH-1015 Lausanne, Switzerland; ^3^ Department of Biochemistry, Indian Institute of Science, Bangalore 560012, India; ^4^ The Francis Crick Institute, 1 Midland Road, London, NW1 1AT, UK; ^5^ Randall Centre for Cell and Molecular Biophysics, School of Basic and Medical Biosciences, King's College London, London, SE1 1UL, UK

**Keywords:** Actin, Live imaging, Tropomyosin, mNeonGreen, Nanobody, Cytokinesis

## Abstract

Tropomyosins are structurally conserved α-helical coiled-coil proteins that bind along the length of filamentous actin (F-actin) in fungi and animals. Tropomyosins play essential roles in the stability of actin filaments and in regulating myosin II contractility. Despite the crucial role of tropomyosin in actin cytoskeletal regulation, *in vivo* investigations of tropomyosin are limited, mainly due to the suboptimal live-cell imaging tools currently available. Here, we report on an mNeonGreen (mNG)-tagged tropomyosin, with native promoter and linker length configuration, that clearly reports tropomyosin dynamics in *Schizosaccharomyces pombe* (Cdc8), *Schizosaccharomyces japonicus* (Cdc8) and *Saccharomyces cerevisiae* (Tpm1 and Tpm2). We also describe a fluorescent probe to visualize mammalian tropomyosin (TPM2 isoform). Finally, we generated a camelid nanobody against *S. pombe* Cdc8, which mimics the localization of mNG–Cdc8 *in vivo*. Using these tools, we report the presence of tropomyosin in previously unappreciated patch-like structures in fission and budding yeasts, show flow of tropomyosin (F-actin) cables to the cytokinetic actomyosin ring and identify rearrangements of the actin cytoskeleton during mating. These powerful tools and strategies will aid better analyses of tropomyosin and F-actin cables *in vivo*.

## Teaser

**Summary:** Visualization of tropomyosin in living yeasts and mammalian cells.

## INTRODUCTION

Tropomyosins are coiled-coil α-helical dimeric proteins that bind and strengthen actin filaments. These proteins are 160–280 amino acids in length (in approximate integer multiples of 40) in which ∼40 amino acids span an actin monomer along the actin filament (Gimona, 2008; Gunning et al., 2015; Holmes and Lehman, 2008). Tropomyosins assemble into unidirectional head-to-tail polymers along an actin filament and this polymerization increases affinity for actin filaments ∼100-fold (Gimona, 2008; Gunning et al., 2015; Holmes and Lehman, 2008). They are widely present in fungal and animal lineages, but clear tropomyosin homologs have not been identified in other phyla or kingdoms (Gunning et al., 1997, 2015; Lin et al., 1997; Martin and Gunning, 2008; Perry, 2001; Pruyne, 2008). In the muscle, tropomyosins regulate actin–myosin II interaction in response to Ca^2+^ release (Brown and Cohen, 2005; Chalovich et al., 1981; Gergely, 1974; Perry, 2001; Spudich and Watt, 1971; Szent-Gyorgyi, 1975; Wakabayashi, 2015). They assemble along with filamentous actin and troponins into ‘thin filaments’, whose interaction with the myosin II motors located in ‘thick filaments’ generates contractile forces. In animal non-muscle cells, tropomyosins play crucial roles in cytoskeletal organization, cell polarity and cytokinesis, and are detected in stress fibers, lamellipodia, the cleavage furrow and the cell cortex (Gunning et al., 2015; Lin et al., 1997). Through work largely in budding and fission yeasts, fungal tropomyosins have been shown to localize to long formin-generated actin cables, the cytokinetic apparatus and the fusion focus, an actin-rich zone observed during mating and fusion of cells of opposite mating types (Alioto et al., 2016; Balasubramanian et al., 1992; Drees et al., 1995; Dudin et al., 2017; Gunning et al., 2015; Huckaba et al., 2004; Liu and Bretscher, 1992; Pruyne, 2008; Skau et al., 2011; Skau and Kovar, 2010; Wloka et al., 2013; Yang and Pon, 2002). Imaging of fixed wild-type cells with antibodies shows that the *S. pombe* tropomyosin also localizes to actin patches (Balasubramanian et al., 1992; Skoumpla et al., 2007). However, none of the available fluorescent protein tools for live imaging detect tropomyosin in patches.

Despite the essentiality of tropomyosins for cell viability and for stabilization of formin-generated actin filaments, investigation of their dynamic properties in live cells are limited. This is largely due to the sub-optimal fluorescent probes available for *in vivo* imaging of tropomyosins, especially in fungi. In this work, we have designed and generated a new mNeonGreen (Shaner et al., 2013) (mNG)–tropomyosin probe that detects actin cables, actomyosin rings, and fusion foci in one or more of three different yeasts (*Schizosaccharomyces pombe*, *Schizosaccharomyces japonicus* and *Saccharomyces cerevisiae*). We also extended this approach to investigate tropomyosin dynamics in mammalian cells, using human RPE cells as an example. Finally, we developed camelid nanobodies against *S. pombe* tropomyosin, encoded by the *cdc8* gene, which expands the toolkit to study tropomyosin function in this yeast and provides a general strategy for the study of *in vivo* dynamics of tropomyosin in other organisms.

## RESULTS

### Live imaging of the mNG-tagged *S. pombe* Cdc8 tropomyosin

**Fig. 1.**
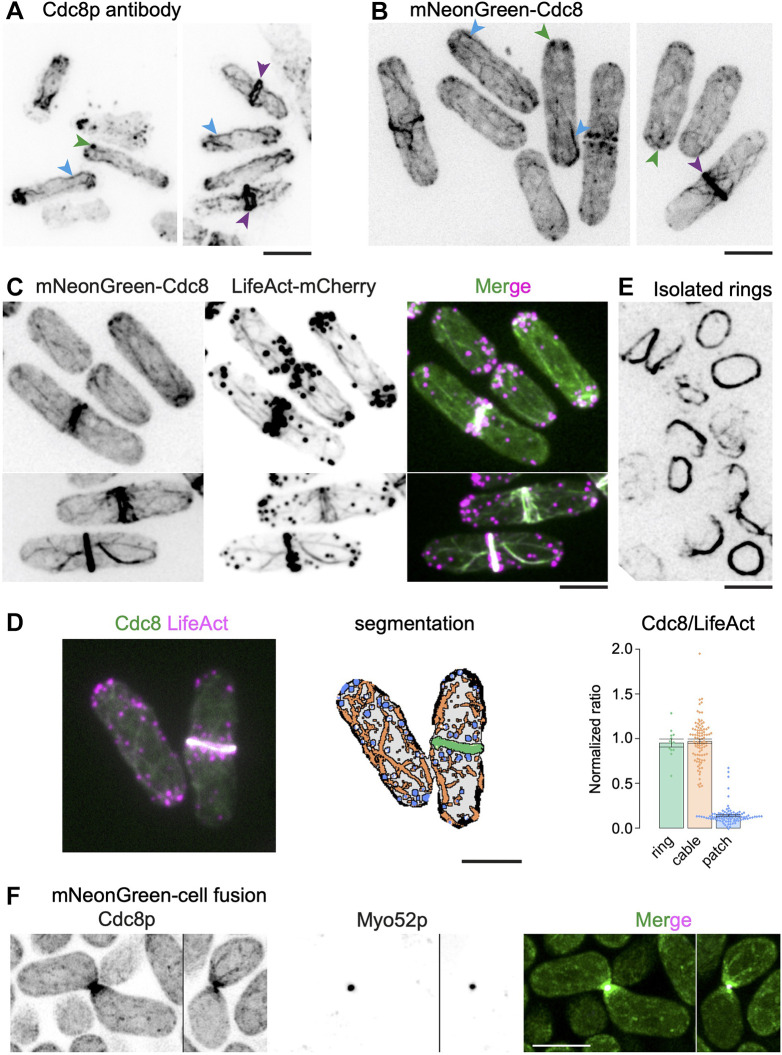
**The tropomyosin Cdc8 localizes to patches, cables, the CAR and the fusion focus in *S. pombe*.** (A) Wild-type cells were fixed and treated with antibodies against Cdc8 to visualize the tropomyosin Cdc8 (*n*=99). (B) Cdc8 was N-terminally tagged with mNG to visualize its localization in patches, cables and the cytokinetic ring. Green arrowheads, mNG–Cdc8 patches; blue arrowheads, mNG–Cdc8 cables; purple arrowheads, mNG–Cdc8 in the CAR (*n*=77). (C) Cells co-expressing mNG-Cdc8 (left) and LifeAct-mCherry (middle) show the co-localization of actin and Cdc8 (*n*=236). A merged fluorescence image shows that whereas actin cables and Cdc8 cables visibly colocalize, only a subset of F-actin patches contained Cdc8 signal. (D) Quantification of relative fluorescence intensity [mean±s.e.m., *n*=13 (ring), 93 (cable) and 79 (patch)] of mNG–Cdc8 versus LifeAct–mCherry in patches, cables, and CARs (right-hand side panel) using automated segmentation of average *z*-projected images. The left-hand side panel shows a typical image used for the automated segmentation, which is shown in the middle panel. (E) Cytokinetic rings were isolated from cells expressing mNG–Cdc8 and the image shows stable association of mNG–Cdc8 with CARs within cell ghosts (*n*=10). (F) Airyscan2 images of mating cells expressing mNG–Cdc8 (green) and Myo52–tdTomato (magenta), which labels the fusion focus. mNG–Cdc8 strongly accumulates at the fusion focus and labels actin cables, which can be seen emanating from the fusion focus and at other positions in the cells. Scale bars: 5 µm.

In *S. pombe*, the *cdc8* gene encodes an essential 161-amino-acid tropomyosin (Balasubramanian et al., 1992). This protein is essential for actin cable stability, cell fusion focus formation and cytokinetic actomyosin ring (CAR) assembly, and thus, for cell mating and cytokinesis (Billault-Chaumartin and Martin, 2019; Christensen et al., 2019; Dudin et al., 2017; Pelham and Chang, 2001; Skau et al., 2009). Consistent with this, staining of wild-type *S. pombe* cells (Balasubramanian et al., 1992; Palani et al., 2019; Skoumpla et al., 2007) with antibodies raised against Cdc8 reveals its localization in cables that run along the long axis of the cell and cytokinetic actomyosin rings (Fig. 1A). Cdc8 is also detected in patches at the cell ends, which in several cases are connected to the actin cables (Skoumpla et al., 2007) (Fig. 1A). Cdc8 has been visualized in live cells as a fusion with a green fluorescent protein (GFP), under the control of an attenuated thiamine-repressible *nmt1* promoter (Billault-Chaumartin and Martin, 2019; Johnson et al., 2014; Maundrell, 1993). GFP–Cdc8 is clearly detected in the CAR, and weakly in actin cables, but is not detected in patches (Pelham and Chang, 2002; Skoumpla et al., 2007) and, therefore, this construct has not been used to investigate patch or cable dynamics.

To facilitate *in vivo* study of tropomyosin dynamics, we therefore set out to make an improved probe. Three parameters [*cdc8* promoter driving fusion gene expression, an mNG fluorescent protein, and a 40-amino-acid flexible linker (Hussain et al., 2018) between mNG and Cdc8] helped generate a fission yeast strain in which we were able to visualize Cdc8 in all three structures (Fig. S1A). Through live imaging of this strain (Fig. 1B), as with antibody staining of fixed cells, mNG–Cdc8 was detected in small patches, actin cables (some of which were connected to actin patches) and the actomyosin ring (which was connected to a meshwork of actin cables). Although expression of mNG–Cdc8 in the presence of native Cdc8 did not cause any overt dominant phenotype (Fig. S2A), as with the previous GFP–Cdc8 probe, it was not fully functional and was unable to sustain life when present as the sole copy. Nevertheless, this experiment confirmed that Cdc8 is a component of actin patches, cables and the CAR.

To investigate whether mNG–Cdc8 colocalized with F-actin, we imaged a strain expressing mNG–Cdc8 and LifeAct–mCherry, as a marker for F-actin (Fig. 1C). As expected, F-actin was detected in patches, cables and CARs, and mNG-Cdc8 colocalized with actin cables and the CAR. Interestingly, although present in multiple small patches, mNG–Cdc8 was present in fewer patches compared with F-actin. Previous work has shown that the tropomyosin Cdc8 competes with the actin filament cross-linker fimbrin for actin binding (Christensen et al., 2017). Consistent with this, in cells lacking fimbrin (*fim1*Δ), mNG–Cdc8 was more obvious in actin patches (Fig. S2B).

To rigorously quantify the relative abundance of mNG–Cdc8 on actin patches, cables and the CAR, we assessed the relative fluorescence intensity of mNG–Cdc8 versus LifeAct–mCherry in CARs (0.95±0.04, *n*=13), cables (0.97±0.02, *n*=93), and patches (0.14±0.01, *n*=79) (mean±s.e.m, see right-hand panel in Fig. 1D) using automated segmentation of average *z*-projected images.

Next, we tested whether mNG–Cdc8 was retained in CARs isolated from *S. pombe* cells, which should facilitate the investigation of tropomyosin (and F-actin) dynamics during CAR constriction *in vitro*. To this end, mNG–Cdc8-expressing cells were spheroplasted and permeabilized with detergent to isolate cell ghosts carrying the CAR. mNG–Cdc8 was stably associated with the CAR held within permeabilized cell ghosts (Mishra et al., 2013) (Fig. 1E). This experiment established that mNG–Cdc8 was not only potentially useful to investigate tropomyosin dynamics in live cells, but also in isolated CARs.

In *S. pombe*, upon nitrogen starvation, cells of opposite mating types polarize and grow towards each other culminating in the formation of the fusion focus, a formin-assembled structure underlying the concentration of secretory vesicles transported by the myosin V Myo52. This is followed by cell–cell fusion, nuclear fusion, meiosis and sporulation. In mating cells (Fig. 1F), mNG–Cdc8 was prominently detected in the fusion focus, where it decorated a larger region than that marked by Myo52. It also decorated longer cables that appeared to emanate from the zone of cell–cell contact, as well as fine speckles.

The mNG–Cdc8 strain generated in this work provided a far superior signal-to-noise ratio compared to the currently available GFP–Cdc8 strains, in which the *GFP-cdc8* fusion gene is expressed under control of the thiamine-repressible *nmt*41/42 promoter directly fused to the coding sequence of the Cdc8 tropomyosin without an intervening linker (Pelham and Chang, 2002) (Fig. S2C). Collectively, these experiments establish that mNG–Cdc8 is a reliable marker that can be used to investigate the dynamics of the tropomyosin Cdc8 in patches, cables, CARs and during cell fusion.

### mNG–Cdc8 is suitable for long-duration time-lapse imaging in vegetative and mating *S. pombe* cells

Having generated the mNG–Cdc8 strain, we tested its usefulness in several time-lapse imaging experiments. Wild-type cells expressing mNG–Cdc8 were imaged every 3 s for ∼30 min using a spinning disk confocal microscope. mNG–Cdc8 did not undergo significant photobleaching over this time and therefore mNG–Cdc8 enabled us to observe tropomyosin dynamics in *S. pombe* at higher time resolution.

**Fig. 2.**
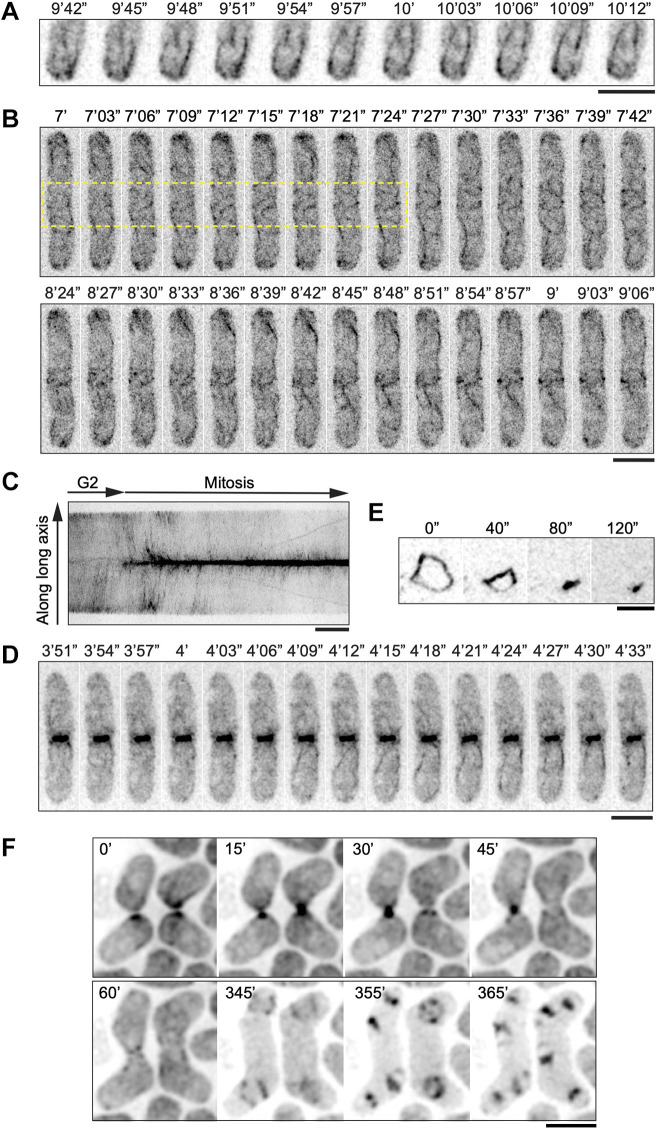
**Visualization of mNG–Cdc8 by time-lapse imaging.** (A) Time-lapse images of wild-type cells expressing mNG–Cdc8 at interphase. Cdc8 exists as patches and cables that originate at the cell tips. Patches can be seen moving on the cables as the cables elongate. (B) Time-lapse images of CAR assembly in wild-type cells expressing mNG–Cdc8. The top panel shows events in a 42 s window in which mNG–Cdc8 assembles into short filaments at the division site (within the yellow box) and into long cables elsewhere. The bottom panel shows movement of non-medial actin cables into the forming CAR. (C) Kymograph of a line parallel to the long axis of a cell transitioning from interphase into mitosis (time, 40 min); mNG–Cdc8 signals found at the cell ends subsequently move towards the cell middle and accumulate to form the CAR. (D) Time-lapse images of wild-type cells during ring constriction in which mNG–Cdc8 cables are seen to be expelled from the CAR. (*n*=82 cell movies for A–D). (E) Time-lapse images showing mNG–Cdc8 behavior in a CAR undergoing constriction within a cell ghost following ATP addition. The CAR constricted in 120 s (*n*=50). (F) Time-lapse epifluorescence images of mating cells expressing mNG–Cdc8. Two pairs of fusing cells are shown, where mNG-Cdc8 decorates the fusion focus (15–45 min), as well as some actin cables before fusion (0 min). The fusion focus disassembles upon fusion (60 min). After the diploid zygote has undergone meiosis, mNG–Cdc8 accumulates on the meiotic actin rings that form during sporulation (345–365 min). Times are defined in minutes (′) and seconds (″). Scale bars: 5 µm.

In interphase cells, multiple small patches of mNG–Cdc8 were detected, some of which showed directional movement from the cell ends towards the cell middle along mNG–Cdc8 cables (Fig. 2A; Movie 1). In mitotic cells, mNG–Cdc8 localized as short filaments at the division site, presumably loading onto Cdc12 (a formin protein)-induced actin filaments assembled from cytokinetic nodes (Vavylonis et al., 2008) (Fig. 2B, top panel; Movie 2). Furthermore, non-medially located mNG–Cdc8 cables were transported to the cell middle where they incorporated into the forming CARs as shown previously for F-actin cables detected using LifeAct–GFP (Huang et al., 2012) (Fig. 2B, bottom panels; Movie 2). To demonstrate the Cdc8 flow toward the cell middle, a kymograph was generated from maximum intensity projected *z*-stack images of a rod-shaped cell by segmentation of the signals from mNG–Cdc8 cables over a line running along the long axis of the cell (Fig. 2C). Transport of non-medially located mNG–Cdc8 cables into the CAR was also observed in highly elongated *cdc25*-22 cells (Fig. S3, Movies 3 and 4) in which these movements were more obvious. In cells undergoing septation, mNG–Cdc8 cables and mNG–Cdc8 aggregates were found to leave the CAR concomitant with its constriction (Fig. 2D; Movie 5). We were also able to observe mNG–Cdc8 in isolated CARs (Fig. 2E), which upon addition of ATP underwent constriction (Movie 6). This is consistent with an earlier study showing that tropomyosin is associated with actin bundles that are expelled during CAR constriction (Huang et al., 2016a).

Next, we tested whether mNG–Cdc8 could be used to investigate tropomyosin dynamics in cells undergoing mating and sporulation. We used epifluorescence imaging to acquire images of mNG–Cdc8 every 5 min for 12 h (Fig. 2F; Movie 7). mNG–Cdc8 localized at growth projections, strongly accumulating at the fusion site, until cell–cell fusion when the signal disappeared, as previously described for the fusion focus (Dudin et al., 2015). A strong mNG–Cdc8 signal re-appeared several hours later, after the diploid zygote had undergone meiosis, with mNG–Cdc8 accumulating on the meiotic actin rings that form during sporulation (Yan and Balasubramanian, 2012).

Collectively, this work established that mNG–Cdc8 is a powerful reporter for time-lapse imaging of dynamics of the tropomyosin Cdc8 in vegetative and sexually reproducing *S. pombe* cells.

### mNG–Cdc8 and mNG–Tpm1 or Tpm2 as tools to visualize tropomyosin in *S. japonicus*, *S. cerevisiae* and human RPE cells

Following the successful visualization of the *S. pombe* tropomyosin Cdc8 using the mNG fusion, we investigated whether the same strategy (promoter and the mNG–40 amino acid linker conjugated to Cdc8, Tpm1 or Tpm2) would lead to visualization of tropomyosin-containing cellular structures in *S. japonicus* and *S. cerevisiae*. Previous work in *S. cerevisiae* has reported Tpm1 and Tpm2 in the CAR and actin cables in fixed cells stained with antibodies (Pruyne et al., 1998), but they had not yet been visualized in cables in live cells. In live *S. japonicus* cells, the 161-amino-acid single tropomyosin has not been visualized.

First, we constructed a *S. japonicus* strain (Fig. S1B) in which the coding sequence for mNG–Cdc8 [P_cdc8_-mNG-40aa linker-cdc8] was integrated into the *ura4* locus as a second copy. As in *S. pombe*, this strain did not show any dominant deleterious growth or division defects, and colony formation rate was comparable to that of wild-type cells (Fig. S4A).

**Fig. 3.**
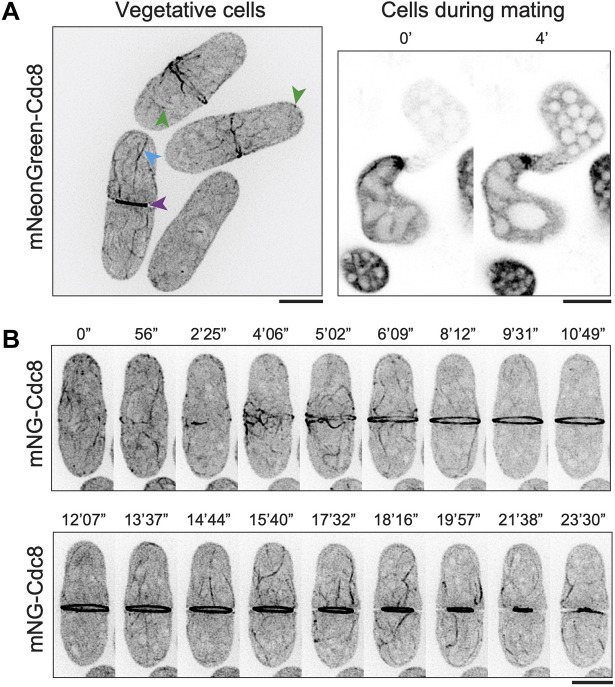
**Visualization of mNG–Cdc8 in *S. japonicus*.** (A) Left, *S. japonicus* cells with mNG–Cdc8 patches (green arrowheads), cables (blue arrowhead) and CAR (purple arrowhead) (*n*=23). Right, Cdc8 patches and cables are seen in *S. japonicus* cells during the mating process (*n*=24). (B) Time-lapse images of CAR assembly and constriction in a *S. japonicus* cell (*n*=24). Note that mNG–Cdc8 assembles at the division site. Longer cables of mNG–Cdc8 also incorporate into the CAR. During constriction, mNG–Cdc8 in cables are expelled from the CAR Times are defined in minutes (′) and seconds (″). Scale bars: 5 µm.

As in *S. pombe*, *S. japonicus* mNG–Cdc8 localized to speckles and/or patches, cables, the CAR and at the fusion focus during mating (Fig. 3A). In time-lapse imaging, mNG–SjCdc8 was detected in non-medially placed cables that were transported and incorporated into the CAR (Fig. 3B; Movie 8). Furthermore, mNG–Cdc8 cables were expelled from the constricting CAR, as seen with F-actin during CAR constriction in *S. japonicus* (Huang et al., 2016a) (Fig. 3B; Movie 8). The incorporation of non-medial mNG–Cdc8 cables into the CAR and its expulsion during CAR constriction were even better resolved in the elongated *cdc25*^ts^ mutant after G2 arrest and release (Fig. S4B; Movie 9). As in *S. pombe*, the mNG–SjCdc8 fluorescence did not bleach rapidly and therefore mNG–SjCdc8 is a useful tool to investigate tropomyosin dynamics in *S. japonicus*.

Next, we constructed mNG-tagged tropomyosin in the budding yeast *S. cerevisiae*, in which the actin cytoskeleton has been extensively characterized. There are two tropomyosin encoding genes in *S. cerevisiae*, Tpm1 (199 amino acids) (Liu and Bretscher, 1989) and Tpm2 (161 amino acids) (Drees et al., 1995; Wang and Bretscher, 1997). Tpm1 abrogation causes loss of detectable actin cables in the cell, which leads to morphological defects. Furthermore, cells lacking Tpm1 and Tpm2 are inviable (Drees et al., 1995), establishing that, as in *S. pombe*, tropomyosin function is essential for *S. cerevisiae* viability. We made the appropriate strains to image Tpm1 and Tpm2, which expressed the appropriate fusion proteins – P_tpm1_-mNG–40 aa linker–Tpm1 (mNG–Tpm1) and P_Tpm2_-mNG–40 aa linker–mNG–Tpm2 (mNG–Tpm2) (Fig. S1C). Cells expressing mNG–Tpm1 and mNG–Tpm2 grew and formed colonies almost identically to wild-type cells (Fig. S5A) and did not show any overt morphological or cell division defects.

**Fig. 4.**
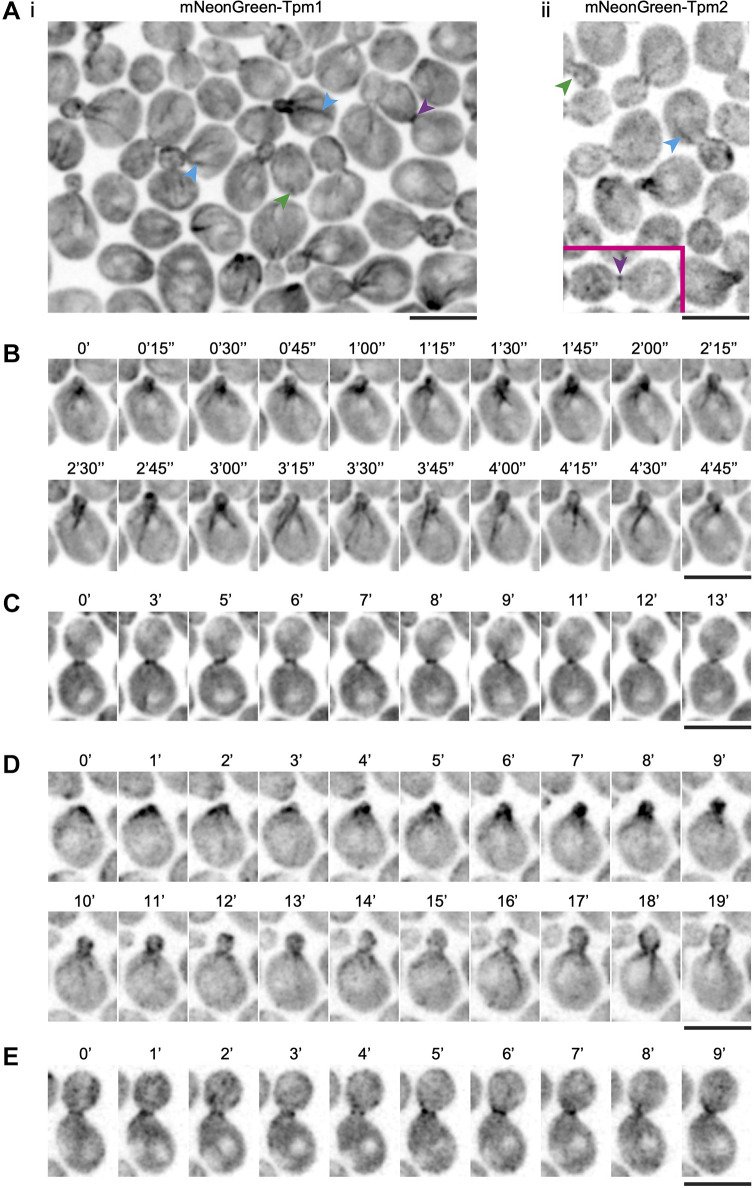
**Visualization of mNG–Tpm1 and mNG–Tpm2 in *S. cerevisiae*.** (A) Images of a field of cells expressing (i) mNG–Tpm1 and (ii) mNG–Tpm2. Patches are indicated with green arrowheads, cables with blue arrowheads and actomyosin rings with purple arrowheads. (B) Time-lapse (min) images revealing the dynamics of mNG–Tpm1 in cables. (C) Time-lapse images of mNG–Tpm1 dynamics during cytokinesis. (D) Time-lapse (min) images revealing dynamics of mNG–Tpm2 in cables. (E) Time-lapse images of mNG–Tpm2 dynamics during cytokinesis. Scale bars: 5 µm. Results representative of *n*=137 cells for Tpm1 and *n*=183 for Tpm2. Times are defined in minutes (′) and seconds (″).

mNG–Tpm1 was detected in patches and in long cables starting near the bud neck and migrating into the mother cell (Fig. 4Ai,B; Movie 10). During cytokinesis, Tpm1 was detected at the division site in the CAR as reported previously (Okada et al., 2021a) (Fig. 4Ai,C; Movie 11). mNG–Tpm2 was detected as a strong focus at the bud site and in cables fainter than those visualized with mNG–Tpm1 (Fig. 4Aii,D; Movie 12); however, it was clearly detected in the CAR (Fig. 4Aii,E; Movie 13). Interestingly, as in *S. pombe*, Tpm1 and Tpm2 were detected in multiple patches in cells in which the gene encoding budding yeast fimbrin, *SAC6*, was deleted (Fig. S5B). Tpm1 and Tpm2 patches were detected in both the mother and daughter cells. This work establishes that mNG–Tpm1 is a reliable tool to visualize Tpm1 in cables and the CAR, and mNG–Tpm2 appears better in the detection of Tpm2 in the CAR, while only weakly detecting Tpm2 in cables.

**Fig. 5.**
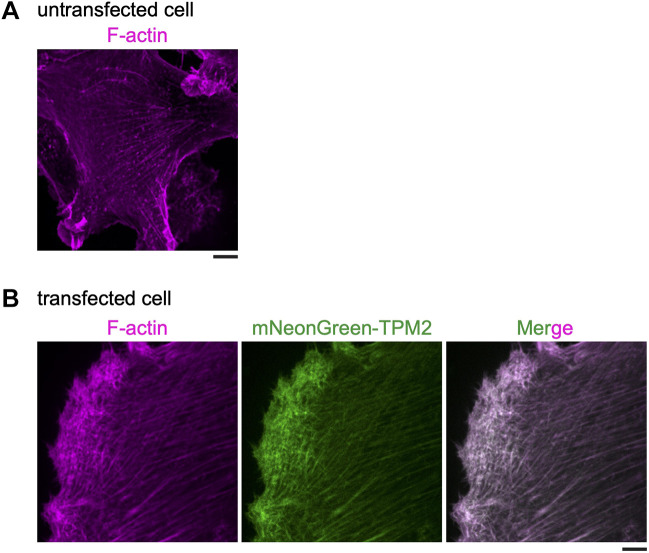
**Visualization of mNG-TPM2 in human RPE cells.** (A). Actin cytoskeleton in an untransfected RPE cell stained with Rhodamine-conjugated phalloidin (*n*=53). (B) Fixed RPE cells expressing mNG-TPM2 show mNG colocalization with Rhodamine–phalloidin-stained F-actin (*n*=43). Scale bars: 5 µm.

Finally, we tested whether the same strategy for visualization of tropomyosin worked in mammalian cells. To this end, we generated a plasmid carrying mNG–40 aa linker–TPM2 (a highly expressed non-muscle tropomyosin isoform splice variant expressing a 284-amino-acid protein), in which expression was driven from the cytomegalovirus (CMV) promoter, and the expressed fusion gene was as follows P_CMV_-mNG–40 aa linker–HsTPM2 (Fig. S1D). In untransfected human RPE cells, F-actin could be found in clear stress fibers with a striated pattern of brighter and weaker alternating signals (Fig. 5A). When the cells were transiently transfected with our plasmid, we were able to detect clear contractile stress fibers with a similar striated pattern, which colocalized with F-actin in fixed cells co-stained with Rhodamine-conjugated phalloidin (Fig. 5A,B), consistent results from with past work (Appaduray et al., 2016; Tojkander et al., 2011; Yang et al., 2020).

These experiments in four organisms/cell types established that mNG–40 aa linker–tropomyosin fusions are a reliable marker to investigate the dynamics of tropomyosin and its associated actin filaments.

### A camelid nanobody faithfully reports the localization of the *S. pombe* tropomyosin Cdc8

In recent years, single domain camelid nanobodies have become powerful tools to investigate protein localization and function (Kirchhofer et al., 2010). These nanobodies are relatively small (∼15 kDa), soluble, and suitable for various light and super-resolution microscopic approaches, including the direct visualization of protein conformational states (Keller et al., 2019; Kirchhofer et al., 2010; Liu et al., 2020). We sought to make nanobodies against *S. pombe* Cdc8 as a proof of concept to test the efficacy of nanobodies in investigating tropomyosin dynamics. To this end, we purified recombinant dimeric Cdc8 tropomyosin, which was used to commercially select interacting nanobodies through phage display screening followed by yeast two-hybrid screening (Moutel et al., 2016). We obtained a panel of seven nanobodies, which were screened through additional yeast two-hybrid screening (Fig. S6A) and by their ability to detect the tropomyosin Cdc8 *in vivo* as N- or C-terminal mNG fusions expressed from one of three promoters (ADH11, ADH21 or ADH81). The entire panel of nanobodies and their full characterization will be reported elsewhere.

**Fig. 6.**
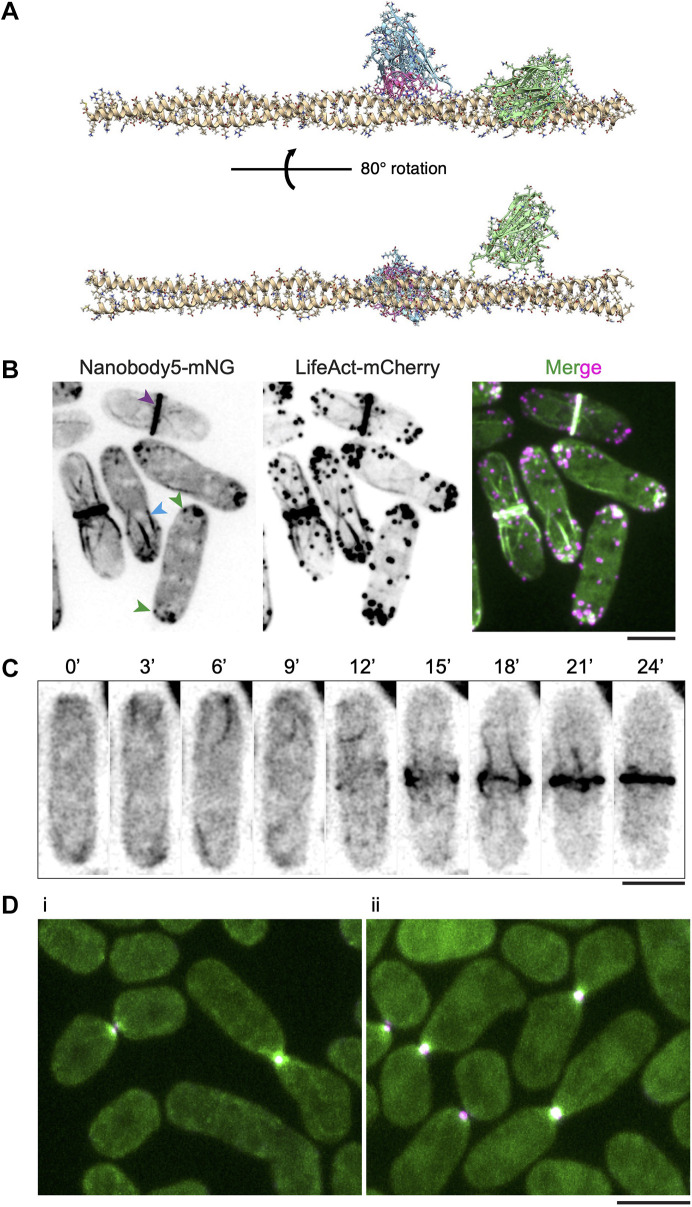
**Visualization of Cdc8 with a targeting-nanobody in *S. pombe*.** (A) Alphafold2 prediction of binding between the dimeric *S. pombe* tropomyosin Cdc8 and Nanobody 5 (Nb5). Nb5 (blue) is predicted to bind, via its complementarity-determining regions (CDRs; shown in pink) to amino acids 86–103 on Cdc8 (golden color dimer). Alphafold2 prediction of a non-specific nanobody targeting a peptide from β-catenin (Braun et al., 2016) (green) is also shown as a control. (B) *S. pombe* cells expressing Nanobody 5 (Nb5) fused to mNG (Nb5–mNG) detects the tropomyosin Cdc8 in patches (green arrowheads), cables (blue arrowheads), and the CAR (purple arrowhead) (*n*=85). (C) Time-lapse images (in seconds) of the dynamics of Nb5–mNG as a marker for the tropomyosin Cdc8 during CAR assembly (*n*=167). (D) Spinning disk microscopy images of *S. pombe* mating cells. (i) Mating cells expressing mNG–Cdc8 (green) and Myo52–tdTomato (magenta). Areas of colocalization appear white. (ii) Mating cells expressing Nb5–mNG (green) and Myo52–tdTomato (magenta). Areas of colocalization appear white (*n*=39 in panel i and 37 in panel ii). Scale bars: 5 µm.

One of the nanobodies (Nanobody 5; Nb5) was efficient in detecting Cdc8 *in vivo* and its expression did not lead to any overt growth defects (Fig. S6B). AlphaFold2 (Jumper et al., 2021; Jumper and Hassabis, 2022) predicted a structure with Nb5 and dimeric Cdc8 forming a complex in which the complementarity-determining regions (CDR) of Nb5 formed a large contact (922 Å^2^; 1 Å=0.1 nm) with both chains of Cdc8 via hydrogen bonds with E89, E92, E94, T97 and R103 of Cdc8 and hydrophobic interactions with L90, L91 and L95 of Cdc8 (Fig. 6A; Fig. S6C). In contrast, a Nb specific for β-catenin (PDB: 5IVO) (Braun et al., 2016) as a control (Fig. 6A; Fig. S6C, Movie 14) was predicted to form a much smaller contact (185 Å^2^) with a different part of the Cdc8 dimer. When Nb5 was expressed as a C-terminal mNG fusion, it bound all structures detected previously using Cdc8 antibodies and the mNG–Cdc8 fusion (Fig. 6B). In interphase cells, Nb5 detected patches and cables that followed the long axes of cells, and in mitotic cells it detected the CAR. In time-lapse studies, Nb5 detected flow of Cdc8 actin cables, and therefore actin cables, from non-medial locations into the CAR (Fig. 6C; Movie 15). Finally, as in the case of mNG–Cdc8, Nb5 also decorated the tropomyosin Cdc8 in the fusion focus, and the signal from mNG–Cdc8 and Nb5–mNG were comparable (Fig. 6D). We note that in mating assays, expression of Nb5 led to mild fusion defects and cell lysis. Collectively, this work provides evidence that the camelid nanobody technology can be successfully applied to the investigation of tropomyosin dynamics.

## DISCUSSION

In this work, we have developed tools to visualize tropomyosins in four different organisms/cell types using an mNG fusion strategy. These yeast strains and plasmids for tropomyosin visualization will be made available to the community. Given that the mNG–40 aa linker–tropomyosin fusion works in four scenarios, we believe this strategy will be transferrable to investigate tropomyosin in other fungi and metazoans. Although tropomyosins are known to be N-terminally acetylated, previous *in vitro* and *in vivo* work has shown that N-terminally tagged tropomyosins are acetylation mimetic in nature and that even tropomyosins that are not acetylated *in vitro* readily incorporate into stress fibers *in vivo* (Brooker et al., 2016; Hitchcock-DeGregori and Heald, 1987; Martin et al., 2010). Thereby, the dynamics of the tropomyosin fusions used in this work are likely reflective of their normal physiological behavior. Although *S. pombe* cells expressing mNG–Cdc8 as their sole copy of Cdc8 were inviable (data not shown), this is not uncommon in that several essential cytoskeletal proteins do not support viability when expressed as the sole tagged copy (e.g. actin, tubulin and ESCRTIII), and are typically expressed as tagged second copies (Chen et al., 2012; Snaith et al., 2010). Nevertheless, study of fusions of these cytoskeletal proteins with various fluorescent proteins have enriched our understanding of the cytoskeleton. We note that, although mammalian TPM2 was expressed transiently in our work, the successful tagging opens the possibility of generating stably expressing cell lines and transgenic gene replacement animals expressing the mNG–40 aa linker–TPM fusion gene under native promoter sequences. Future work should evaluate how the mNG–TPM2 fusion we describe compares with those described elsewhere in side-by-side experiments (Appaduray et al., 2016; Tojkander et al., 2011; Yang et al., 2020).

We have also described a workflow, and proof-of-concept in *S. pombe*, by developing nanobodies for the tropomyosin Cdc8, an mNG-tagged version of which we used to visualize tropomyosin *in vivo*, thereby expanding the tool kit to image tropomyosin. In addition to directly developing nanobodies against tropomyosin from other organisms as a primary strategy, an attractive additional possibility is to ‘evolve’ the *S. pombe* tropomyosin nanobodies (based on similarity to the binding epitope) to identify Nb5 variants that recognize tropomyosin from other organisms. We have determined that Nb5 potentially binds amino acids 86–103 on Cdc8 (the molecular characterization of this interaction and those of the other six nanobodies with Cdc8 and other tropomyosins will be reported elsewhere).

### What new insights have we gained and what more can be addressed using these new tools?

First, although fungal tropomyosins have been studied for their roles in actin cables and the CAR, here we have shown, using our mNG–40 aa linker–tropomyosin fusions, that they localize to patches in three wild-type yeast species. A previous study demonstrated Cdc8 localization in patches at the cell ends in live fimbrin-defective mutants (Christensen et al., 2017), although Cdc8-decorated patches have been observed in wild-type cells using immunofluorescence microscopy on fixed and permeabilized cells. The fact that the tropomyosin Cdc8 was detected in patches in live wild-type *S. pombe* cells using two different probes established that the tropomyosin Cdc8 might only be partly out-competed by fimbrin and might have a function in patches under normal physiological conditions. This localization mirrors the weak, occasional localization of the formin For3 at actin patches, which is mostly outcompeted from patches by capping proteins (Billault-Chaumartin and Martin, 2019). Cdc8 and other tropomyosins in patches might thus represent the imperfect sorting of actin-binding proteins. However, the presence of the tropomyosin Cdc8 only in a subset of actin patches suggests that it might alter or regulate actin patch function, perhaps by playing important temporal roles in patch biogenesis and function. The localization of mNG–Tpm1 and mNG–Cdc8 to patches in *S. cerevisiae* and *S. japonicus*, respectively, raises the possibility that tropomyosin might have functional roles in actin patches in other fungi as well. It is also possible that formins use severed actin filaments from actin patches to elongate linear actin cables and the patches might represent sites from which tropomyosins are loaded onto actin filaments. The dynamics and function of tropomyosin in actin patches and how this relates to other actin patch components and endocytosis, as well as how it relates to formins and actin cable elongation, can now be studied using the tools we have generated.

Second, previous work on CAR assembly has used non-physiological probes to visualize CAR F-actin assembly (such as CHD, Utrophin or LifeAct), which influence the kinetics of F-actin polymerization and depolymerization (Huang et al., 2012; Vavylonis et al., 2008). Therefore, it has been debated whether the F-actin non-medial flow toward the ring was physiological. mNG–Cdc8 is observed in *de novo* generated filaments at the division site and in non-medial cables that are transported to the CAR in *S. pombe*. These signals are lost upon treatment with the actin polymerization inhibitor latrunculin A (Ayscough et al., 1997), establishing that the mNG–Cdc8 can be a valuable tool to investigate actin cables. Our observation of the dynamics of the tropomyosin Cdc8 provides direct evidence for two mechanisms in operation during CAR F-actin assembly, namely, direct F-actin nucleation at the cell division site from cytokinetic nodes, and transport of non-medial F-actin cables. The rate of assembly, the extent of turnover, and the velocity of cable movement can now be investigated. The ability to use the superior mNG–Cdc8 in CARs in cell ghosts also allows the investigation of Cdc8 and actin filament dynamics during myosin-II-dependent CAR constriction (triggered by ATP addition to isolated CARs).

Third, mNG–Cdc8 provides superior imaging of the actin cytoskeleton during mating and meiosis. Although Cdc8 had previously been described at the fusion focus (Dudin et al., 2017; Kurahashi et al., 2002), mNG–Cdc8 reveals a more extensive signal that extends over a larger zone than that occupied by the type V myosin Myo52, and in particular shows a better signal-to-noise ratio compared with the previous GFP–Cdc8 probe. Our investigations also revealed a function for tropomyosin in meiotic actin rings (Yan and Balasubramanian, 2012), in which the tropomyosin Cdc8 had not been previously detected. Because For3 cooperates with the Arp2/3 complex to assemble the meiotic actin rings (Yan and Balasubramanian, 2012), this suggests that Cdc8 decorates and perhaps stabilizes the For3- and formin-assembled part of the meiotic actin rings.

Fourth, tools composed from native proteins are not available to investigate dynamics of actin cables and the CAR in *S. japonicus*. Despite close phylogeny and morphological similarity to *S. pombe*, CAR assembly in *S. japonicus* is executed through a different, post-anaphase mechanism (Gu et al., 2015). Currently, the proportion of medial CAR actin filaments derived from medial ‘node-dependent’ nucleation versus those transported from non-medial locations is unknown. This can now be investigated with the tools developed in this work.

Finally, although extensive work has been done on *S. cerevisiae* actin cables (Alioto et al., 2016; Chesarone et al., 2009; Huckaba et al., 2004; McInally et al., 2021; Miao et al., 2016, 2013; Yang and Pon, 2002), the dynamics of Tpm1 or Tpm2 in interphase cables has not been explored in live cells. Furthermore, an extensive body of work has been done on the interplay between actin patches, actin cables and endocytosis (Drubin et al., 2005; Kaksonen et al., 2005, 2006). The mNG–Tpm1 and mNG–Tpm2 strains should allow further characterization of such links between actin patches, cables, and endocytosis. The mNG–Tpm1 and –Tpm2 tool can also be used to determine isoform specific localization patterns for Tpm1 and Tpm2, which would help in distinguishing their shared and distinct functions with respect to the *S. cerevisiae* actin cytoskeleton. We note that although mNG–Tpm1 and mNG–Tpm2 show a similar distribution, the intensity of Tpm2 in various structures is weaker and more prone to photobleaching compared to Tpm1. In the future, we intend to make fusions of Tpm2 with the far superior and photostable Stay Gold protein (Hirano et al., 2022). Such an analysis will clarify whether Tpm1 and Tpm2 differ in their distribution and/or dynamics.

## MATERIALS AND METHODS

### Yeast strains and plasmids

All yeast strains and plasmids used in this study are listed in Table S1 and Table S2, respectively.

### Construction of the *S. pombe* integration plasmid pDUAL:Pcdc8:mNeonGreen-40aa:cdc8 to express mNeonGreen–Cdc8

To isolate Fragment 1, the sequence of mNeonGreen fused with a 40-amino-acid flexible linker sequence (40 aa linker; LEGSGQGPGSGQGSGSPGSGQGPGSGQGSGPGQGSGPGQG) was PCR amplified from plasmid pDUAL-Padh21-mNeonGreen-40aa, #TH-8-76 (Balasubramanian laboratory plasmid collection) using a pair of PCR primers (forward primer, 5′-ATGGTGAGCAAGGGCGAGG-3′; reverse primer, 5′-TCCCTGACCGGGGCC-3′). To isolate Fragment 2, the pDUAL vector expressing Cdc8 under the control of the native *cdc8* promoter and terminator (used in Palani et al., 2019) was PCR amplified in its entirety using a pair of primers (forward primer: 5′-GTTCTGGCCCCGGTCAGGGAATGGATAAGCTTAGAGAGAAAATTAATGCCGC-3′; reverse primer: 5′-TCCTCGCCCTTGCTCACCATTTTCCTACTGTTTCCTTCTTTCCTTGATGG-3′). The resulting linear DNA fragment contained the *cdc8* coding sequence at the 5′ end and the *cdc8* promoter sequence (Pcdc8) at the 3′ end.

pDUAL:Pcdc8:mNeonGreen-40aa:cdc8 (denoted plasmid #TH8-77) was constructed by Gibson assembly of fragments 1 and 2. Using 20 bp overlapping sequences between the ends of the fragments, the *cdc8* and Pcdc8 ends of fragment 2 were fused to the linker and mNG ends of fragment 1, respectively.

Plasmid #TH8-77 was digested with the NotI restriction enzyme and the DNA fragment containing the *cdc8*-mNG-40-aa-linker sequence was purified by gel extraction (Qiagen). The fragment was used for the transformation of 968 h^90^ derived strains MBY101 (*h-*, *leu1-32*, *ura4-D18*, *ade6-210*) and MBY102 (*h+, leu1-32*, *ura4-D18*, *ade6-210*) by using a lithium acetate based method (Alfa et al., 1993). The DNA fragment was integrated at the *leu1-32* gene locus of the cells by endogenous homologues recombination as described previously (Matsuyama et al., 2008). The *leu1^+^* gene was reconstructed on the resulting transformants to produce leucine prototrophy, and the new strains, MBY12825 (h-) and MBY12828 (h+), were selected on Edinburgh minimal medium (EMM; Petersen and Russell, 2016) containing uracil, adenine, lysine and histidine. For the spot assay, *S. pombe* cells were cultured in YEA medium at 24°C to saturation, subjected to six tenfold serial dilutions, and 5 μl aliquots of each dilution spotted onto yeast extract with adenine (YEA) agar (Petersen and Russell, 2016). Plates were incubated at 24°C and 36°C for 3 days before being photographed.

### Generation of mNG-tagged *S. japonicus* tropomyosin

To express a N-terminus fluorescent protein-tagged *S. japonicus* Cdc8 tropomyosin (SJAG_04887) as a second copy in *S. japonicus* cells, a DNA fragment of mNeonGreen–40-amino-acid linker–Cdc8 was synthesized and cloned into pUC-GW-Kan (Azenta Life Sciences) by GeneWiz. This fragment contains the 5′ UTR of *cdc8* between GGGTTTAGTGAG and CAAGAACATCAA, followed by the open reading frame of mNG, a 40-amino-acid peptide linker (LEGSGQGPGSGQGSGSPGSGQGPGSGQGSGPGQGSGPGQG), the coding sequence of *cdc8*, and the 3′ UTR of *cdc8* ending at TGTCTTGCTTAG. The DNA fragment of mNG–40 amino acid linker–cdc8 was subsequently cloned into pSO550 (Gu et al., 2015) flanked by Kpn I and BamHI restriction enzyme sites. The pSO550-mNG–40 amino acid linker–cdc8 plasmid was linearized by using the restriction enzyme AfeI and integrated into the genome of an NIG2021-derived *S. japonicus* strain at the *ura4sj-D3* locus according to a previously described electroporation protocol (Aoki et al., 2010).

### Generation of *S. cerevisiae* mNG–Tpm1- and mNG–Tpm2-expressing strains

Genomic DNA of strain ESM356 (wild-type) was isolated using a lithium acetate-SDS based protocol as previously described (Looke et al., 2011). PCR amplification of the desired fragments was performed using NEB Q5 High-Fidelity DNA polymerase (#M0491S, NEB). The PCR fragments were assembled into the linearized vector pRS305 (integration vector; Sikorski and Hieter, 1989) using NEBuilder HiFi DNA Assembly reaction mix (#E2621L, NEB) as per the manufacturer's instructions. The reaction product was transformed into *Escherichia coli* TOP10 cells (#C404010, Invitrogen) and plasmid isolation was performed using the Thermo Fisher GeneJet Miniprep Kit (#K0503, ThermoScientific). The positive transformants were confirmed using restriction digestion and sequencing. ESM356 (S288c background) was used as a wild-type strain and all subsequent strains were derived from it. Yeast culturing and transformation were undertaken using established protocols. Yeast expression plasmids carrying mNG–Tpm1 and –Tpm2 (piSP347 and piSP349, respectively) were linearized with the KasI restriction enzyme and transformed for integration into the *leu2* locus in the yeast genome as a second copy. The positive transformants were selected on SC-Leu plates and expression of mNG–Tpm1 and –Tpm2 were confirmed using fluorescence microscopy.

### Generation of mNG–TPM2-expressing RPE-1 cells

For the mNG–TPM2 mammalian expression plasmid construction, a mammalian expression backbone vector for humanized mNG, pmNeonGreenHO-G (Addgene #127912), was linearized using BspE I restriction enzyme at 37°C. A double stranded DNA gblock (IDT) was synthesized consisting of a 40-amino-acid flexible linker region (LEGSGQGPGSGQGSGSPGSGQGPGSGQGSGPGQGSGPGQG) fused to the coding sequence of the N-terminus of the human tropomyosin 2 (TPM2) protein (NCBI, NP_003280.2). Overhang sites complementary to the vector were added to both gblock ends via PCR (forward primer, 5′-GGCATGGACGAACTCTATAAGCTGGAAGGCTCTGGCCAGGGT-3′; reverse primer, 5′-ACCGCCTCC ACCGGATCTGAGTTACAAGGATGTTATATCATT-3′) and the fragment was cloned into the vector using NEBuilder^®^ HiFi DNA Assembly Master Mix (NEB, #E2621L) to generate a sequence encoding mNG–40 aa linker–TPM2 under control of a CMV promoter. Successful cloning was confirmed via sequencing (forward primer, 5′- CCGGACAATGCAGTTTGAAG-3′) and restriction enzyme digestion.

Immortalized (hTERT) diploid human retinal pigment epithelial (RPE1) cells (ATCC; CRL-4000) were cultured in Dulbecco's modified Eagle's medium with nutrient mixture Ham's F-12 with 15 mM HEPES and sodium bicarbonate (Sigma, D6421) supplemented with 6 mM L-glutamine (Gibco, 25030-081), 10% fetal bovine serum (Sigma, F7524), 100 U/ml penicillin and 100 µg/ml streptomycin (Gibco, 15140-122) at 37°C under 5% CO_2_.

For transfection, RPE-1 cells were grown on ibiTreat 2-Well µ-Sildes (Ibidi, 80286) to 80% confluency. Cells were then transfected using Lipofectamine® 2000 (Invitrogen, 11668-019), and Opti-MEM^®^ reduced-serum medium (Gibco, 31985-062) according to the manufacturer's instructions.

### Live-cell imaging

#### S. pombe

An Andor Revolution XD spinning disk confocal microscopy was used for image acquisition. The imaging system was equipped with a Nikon ECLIPSE Ti inverted microscope, Nikon Plan Apo Lambda 100×/1.45 NA oil-immersion objective lens, a spinning disk system (CSU-X1; Yokogawa Electric Corporation), and an Andor iXon Ultra EMCCD camera. Images were acquired using the Andor IQ3 software at 69 nm/pixel. The fluorophores were excited by laser lines at wavelengths of 488 or 561 nm. All images were acquired at 25°C.

For immunofluorescence, *S. pombe* cells were grown in YEA medium at 24°C to mid-log phase [optical density at 595 nm (OD_595_) of 0.2–0.4]. Cells from 20 ml of culture were collected by centrifugation at 956 ***g*** for 2 min, washed with PBS, fixed in 4% paraformaldehyde at room temperature for 12 min, washed again with PBS, and resuspended in 200 µl of protoplasting solution. Protoplasting solution consisted of 1 mg/ml lysing enzyme (Sigma, L1412) and 6 µl/ml Zymolyase (GBiosciences, 1.5 U/µl) in PBS with 1.2 M sorbitol. The cells were incubated at room temperature for 15 min and visually checked for protoplasting by mixing an aliquot with 10% SDS at a 1:1 ratio. To inactivate the protoplasting enzymes, 1 ml of 1% Triton X-100 was added and incubated for 2 min. Cells were then spun down (450 ***g*** for 1 min), blocked by resuspension in 0.5 ml PBAL (10% BSA, 10 mM lysine hydrochloride, 50 ng/ml carbenicillin and 1 mM sodium azide), and incubated at room temperature for 1 h with gentle rocking. After centrifugation (450 ***g*** for 1 min), a primary, affinity-purified anti-Cdc8p antibody was added (1:200 in PBAL; Balasubramanian et al., 1992) and the mix was incubated at 4°C overnight. Cells were subsequently washed twice in PBAL. A secondary antibody (Life Technologies, 1420898) was added (1:200 in PBAL) for 90 min at room temperature. Following two washes, the cells were ready for imaging.

To prepare samples for live-cell imaging, *S. pombe* cells were grown in YEA medium at 24°C to mid-log phase (OD_595_ 0.2–0.4). Cells from 20 ml of culture were collected by centrifugation at 450 ***g*** for 1 min and resuspended in a small volume of YEA sterilized by filtration. A small aliquot was then added onto a 2% agarose in YEA medium pad, covered with a coverslip, and the edges sealed with VALAP.

For Fig. 2A–C,E, time-lapse images were acquired at 3 s intervals. A total of 16 planes were imaged with *Z*-step sizes of 0.4 µm. For Fig. 4C, time-lapse images were acquired at 1 min intervals. A total of 13 planes were imaged with *Z*-step sizes of 0.5 µm. For Fig. S3, *S. pombe cdc25-22* cells were grown in YEA medium at 24°C to mid-log phase (OD_595_ 0.2–0.4) and then blocked at the G2/M transition by incubating them at 36°C for 4 h. The cells from 20 ml of culture were then prepared for imaging as described in the above paragraph. Time-lapse imaging commenced about 15 min after the cells were moved from 36°C into 25°C for synchronous release into the cell cycle. Time-lapse images were acquired at 2 min intervals, and a total of 17 planes were imaged with *Z*-step sizes of 0.5 µm.

Live microscopy of strain MBY12947 (Fig. 1D) was performed using an Andor TuCam system equipped with a Nikon ECLIPSE Ti inverted microscope, a Nikon Plan Apo Lambda 100×/1.45-NA oil-immersion objective lens, a spinning-disk confocal system (CSU-X1; Yokogawa Electric Corporation), two Andor iXon Ultra EMCCD cameras, and a wavelength filter set consisting of a 561-nm single-edge laser-flat dichroic beamsplitter, a 514/30-nm single-band bandpass filter, and a 568-nm ultrasteep long-pass edge filter (Semrock). Simultaneous image acquisition of fluorophores excited by laser lines at wavelengths of 488 nm and 561 nm was executed by Andor IQ3 software. A total of 17 planes were imaged at an exposure time of 100 ms, 0.5 µm *z*-steps, and at 69 nm/pixel. Subtle image misalignment between the two channels was digitally corrected by using a custom MATLAB script kindly provided by Dr Darius Koester (Division of Biomedical Sciences, University of Warwick, UK).

#### *S. pombe* CAR isolation and *in vitro* constriction

*S. pombe* cells were grown in YEA medium at 24°C to mid-log phase (OD_595_ 0.2–0.4). The cells from 20 ml of culture were collected by centrifugation at 956 ***g*** for 1 min, washed with an equal volume of 1× E-buffer (50 mM sodium citrate and 100 mM sodium phosphate, pH6.0) and resuspended in 5 ml of 1× E-buffer supplemented with 1.2 M sorbitol, to which 0.6 mg/ml of lysing enzyme (Sigma, L1412) was added. Cells were incubated at 24°C with shaking at 80 rpm. After 1.5 h, 30 µl of LongLife Zymolyase (GBiosciences, 1.5 U/µl) was added, and the cells were incubated at 24°C for another 1.5 h. The following washes were done with centrifugation at 450 ***g*** for 2 min. Protoplasts were washed with 1× E-buffer with 0.6 M sorbitol and then resuspended in EMM supplemented with 0.8 M sorbitol. The protoplasts were incubated at 24°C with shaking at 80 rpm for 3.5 h. The following steps were done on ice. After a wash with wash buffer, cell ghosts were obtained by permeabilizing the protoplasts with isolation buffer containing 0.5% NP-40. The cell ghosts were then homogenized with a glass homogenizer to obtain isolated rings. The rings were washed and resuspended in reactivation buffer, and imaging of the isolated rings was undertaken using a CellASIC ONIX Microfluidic system (Merck Millipore) with 0.5 mM ATP in reactivation buffer used to induce ring constriction. Please note all buffers were as described previously (Huang et al., 2016b).

Images were viewed and analyzed using ImageJ. Unless otherwise indicated, all image stacks were projected along the *Z* axis (maximum intensity) for analysis and for representation. If applicable, the movies were bleach corrected using ‘Image/Adjust/Bleach Correction’. The brightness and contrast of all microscopy images was adjusted by using ‘Image/Adjust/Brightness & contrast’. All time-lapse videos were edited in ImageJ and saved in MP4 format with H.264 compression.

To quantify the signals of LifeAct and Cdc8 on the actin structures, we performed automated segmentation of average *z*-projected images with the Trainable Weka Plugin in Fiji/ImageJ. We trained classifiers to detect actin patches, the cytoplasm (regions in a cell without specific actin structures) and the outside of the cells with the LifeAct images and to detect actin cables and actin rings with the Cdc8 images. From 12 two-channel images, 93 separate cells or a pair of daughter cells still connected were detected and analyzed further (six clumps of cells were omitted). The generated masks for these objects were converted into regions of interest (ROIs) per cell, and the area and mean intensities for both the channels were measured with a custom ImageJ macro. To strictly separate the actin cables from the cytoplasm, the ROIs for the actin cables were shrunk by 1 pixel and the 10% darkest pixels in the actin structures and 10% brightest pixels in the cytoplasm were omitted for the measurement of the mean intensity. Then, the ratio between the mean Cdc8 and LifeAct intensities in a ROI above their respective cytoplasm levels was calculated and normalized for the difference in their expression levels, i.e. the overall signals in the same cell above the outside background signals.

For *S. pombe* mating and sporulation studies, homothallic (h90) strains able to switch mating types were used. Cells were grown in liquid or agar minimum sporulation media (MSL), with or without nitrogen (+/− N) (Egel et al., 1994). The protocol for live imaging of *S. pombe* mating cells was adapted from Vjestica et al. (2016). Briefly, cells were first pre-cultured overnight in MSL+N at 25°C, then diluted to OD_600_=0.05 into MSL+N and incubated at 25°C for 20 h. Exponentially growing cells were then pelleted, washed with MSL-N by three rounds of centrifugation, and resuspended in MSL–N to an OD_600_ of 1.5. Cells were then grown for 3 h at 30°C to allow mating in liquid, added onto 2% agarose MSL–N pads, and covered with coverslips whose edges were sealed with VALAP. We allowed the pads to rest for a minimum of 30 min at 30°C before imaging.

Images presented in Fig. 1F and Fig. S2 were obtained using a ZEISS LSM 980 scanning confocal system equipped with four confocal detectors (2× GaAsP, 2× PMT), an Airyscan2 detector optimized for a 60×/1.518 NA oil objective, and 6 laser lines (405 nm, 445 nm, 488 nm, 514 nm, 561 nm and 640 nm) on an inverted Microscope Axio Observer 7. Images were acquired using the Airyscan2 detector and processed with the Zen3.3 (blue edition) software for super resolution.

Images presented in Fig. 2F were obtained using a DeltaVision platform (Applied Precision) composed of a customized inverted microscope (IX-71; Olympus), a UPlan Apochromat 100×/1.4 NA oil objective, a camera (CoolSNAP HQ2; Photometrics or 4.2Mpx PrimeBSI sCMOS camera; Photometrics), and a color combined unit illuminator (Insight SSI 7; Social Science Insights). Images were acquired using softWoRx v4.1.2 software (Applied Precision). Images were acquired every 5 min for 12 h. To limit photobleaching, overnight videos were captured by optical axis integration (OAI) imaging of a 4.6 μm *z*-section, which is essentially a real-time *z*-sweep.

Images presented in Fig. 6D were obtained using a spinning disk microscope composed of an inverted microscope (DMI4000B; Leica) equipped with an HCX Plan Apochromat 100×/1.46 NA oil objective and an UltraVIEW system [PerkinElmer; including a real-time confocal scanning head (CSU22; Yokagawa Electric Corporation), solid-state laser lines, and an electronmultiplying charge coupled device camera (C9100; Hamamatsu Photonics)]. Images were acquired using the Volocity software (PerkinElmer).

#### S. japonicus

Cells expressing mNG–Cdc8 were grown in yeast extract with supplements (YES) medium (Petersen and Russell, 2016) to an OD_595_ of 0.4-0.6 at 30°C. Prior to imaging, 1 ml yeast culture was concentrated to 50 µl after centrifugation at 1500 ***g*** for 30 s. 1 µl cell suspension was loaded on YES medium on an agarose pad (2% agarose) and covered with a 22×22 mm glass coverslip (VWR #631-0125, thickness: 1.5). Time-lapse imaging of mNG–Cdc8 cells was performed at 30°C. A total of 16 focal planes were imaged, with 0.6 µm *z*-steps, and at 11.2 s time intervals.

Temperature-sensitive *cdc25-D9* cells expressing mNG–Cdc8 were grown in YES medium to an OD_595_ of 0.1 at 24°C. 50 ml of cell culture was incubated at 36°C for 3 h 15 min to block cell cycle progression of these cells at the G2/M transition before the block was removed by incubation at permissive temperature, allowing synchronized mitotic entry of the cells. Time-lapse imaging was performed at 25°C. A total of 13 focal planes were acquired, with 0.6 µm *z*-steps, and at 30 s time intervals.

*S. japonicus* cells expressing mNG–Cdc8 were grown to exponential phase at 30°C in EMM liquid medium that was adequately complemented, then washed in SPA liquid medium by three rounds of centrifugation (956 ***g*** for 5 min), added onto 2% agarose SPA pads, and covered with coverslips whose edges were sealed with VALAP. Cells were then allowed to mate for 7 h before imaging.

Spinning disk confocal images were acquired with a system comprising an iXon Ultra U3-888-BV monochrome EMCCD camera (Andor Technology Ltd., UK), an Eclipse Ti-E inverted microscope (Nikon, Japan) fitted with a CSU-X1 spinning disk confocal scanning unit (Yokogawa electric, Japan), a 600 series SS 488 nm, 50 mW laser, a Brightline single band filter FF01-525/50-25 (Semrock, USA), and a CFI Plan Apo Lambda 100× (N.A.=1.45) oil objective (Nikon, Japan). Image acquisition and deconvolution were controlled by Andor Fusion software (2.3.0.36).

#### S. cerevisiae

Glass bottom dishes (35 mm; #81218, Ibidi GmBH, Germany) were coated with 6% concanavalin A (#C2010, Sigma). Yeast cells grown to mid-log phase in filter-sterilized SC-complete medium (Okada et al., 2021b) at 30°C were spotted on the coated dishes for imaging. Time-lapse imaging was performed at 30°C using an Andor Revolution XD spinning disk confocal microscope. The microscope was equipped with a Nikon ECLIPSE Ti inverted microscope, a Nikon Plan Apo Lambda 100×/1.45-NA oil-immersion objective lens, a spinning disk system (CSU-X1; Yokogawa Electric Corporation), and an Andor iXon Ultra EMCCD camera. A laser line at a wavelength of 488 nm was used for fluorophore excitation. Images in Fig. S5B were acquired using an Andor Dragonfly 502 spinning disk confocal system (Andor Technology Ltd., Belfast, UK) equipped with an Andor Sona scMOS camera mounted on a Leica Dmi8 inverted fully motorized microscope (Leica, Wetzlar, Germany). A total of 18 focal planes were imaged, acquired at 0.35 µm *z*-steps, and using a 100× oil objective (1.4 NA) and a 488 nm solid-state laser for excitation of mNG. Images were deconvolved using Andor Fusion software (2.3.0.44) and processed offline using Fiji (Schindelin et al., 2012).

#### Human RPE cells

Images were acquired with the same microscopy system as the *S. pombe* images (see above), but with a Nikon Plan Fluor 40×/1.30 oil immersion objective lens. Images were acquired at 80 nm/pixel and fluorophores were excited by 488 nm or 561 nm laser lines.

Live-cell imaging of transfected RPE-1 cells was performed in phenol-free Leibovitz's L-15 Medium (Gibco, 21083-027) at 37°C. For Rhodamine–phalloidin fixed-cell imaging, transfected RPE-1 cells were fixed in 4% paraformaldehyde in PBS, stained with Rhodamine–phalloidin (Invitrogen, R415) diluted 1:400 in 0.1% Triton X-100/PBS and sealed with Vectashield^®^ (Vector, H-1000). Images were acquired at room temperature.

### Tropomyosin nanobody construction

The sequence encoding *S. pombe* tropomyosin (SpCdc8) was cloned into the pET^MCN^ vector (without any tag) for protein expression as described previously (Palani et al., 2019). The purified recombinant SpCdc8 was dialyzed against the tropomyosin storage buffer (50 mM NaCl, 10 mM imidazole, pH 7.5, and 1 mM DTT), flash frozen in liquid nitrogen, and sent to Hybrigenics to raise nanobodies against it. The nanobody protein sequences provided by Hybrigenics were codon optimized for *S. pombe* expression and gBlocks were synthesized (IDT, USA). The nanobody gBlocks were cloned into the *S. pombe* vector pDUAL (Matsuyama et al., 2008) under the constitutive expression promoter pADH11, in fusion with mNG along with 40 aa linker at the N-terminus of the nanobodies. Yeast expression nanobody plasmids were linearized with the NotI restriction enzyme and transformed into *S. pombe* strain MBY192 for integration. Colonies were selected on EMM−Leu plates and nanobody expression was confirmed by fluorescence microscopy. The amino acid sequence of the nanobody used in the imaging experiments (Nanobody 5/Nb5), with the CDRs underlined, is:

MAEVQLQASGGGFVQPGGSLRLSCAASGRTYEQSAMGWFRQAPGKEREFVSAISRNSGQWQYYADSVKGRFTISRDNSKNTVYLQMNSLRAEDTATYYCARAFDVLIKGATYSREYWGQGTQVTVSS.

### Yeast two-hybrid experiments

The gene of interest, tropomyosin (SpCdc8) from *S. pombe* genomic DNA, and *S. pombe* codon-optimized nanobodies (A5, A10, A19, A22, A37, A83 and A94) from IDT gBlocks were amplified and cloned into pMM5S (containing the activation domain of Gal4p) and pMM6S (DNA-binding protein LexA) plasmids, respectively (Table S2). Plasmids carrying the genes of interest (SpCdc8 and the seven nanobodies) and empty vector controls were transformed into the S288c-derived yeast strains SGY37 (MATa) and YPH500 (MATα). Transformants with the desired plasmids were selected on SC plates lacking histidine (−His) or leucine (−Leu), respectively. Mating of the MATa and MATα strains was done on YPD plates which were incubated for 2 days at 30 °C, followed by replica-plating onto double selection SC−His−Leu plates. The plates were incubated for 2 days at 30°C before the X-Gal (#RC-212, G-Biosciences, USA) overlay. The X-Gal overlay assay was performed as previously described (Geissler et al., 1996). Plates were scanned after 24 h of incubation with the X-Gal overlay mixture.

### AlphaFold2 prediction

For prediction of the Cdc8-nanobody complex structure, two Cdc8 (CAA93291.2) and one Nb5 sequences or two Cdc8 and one BC2-nanobody (PDB: 5IVO) (Braun et al., 2016) sequences were input into AlphaFold Colab (https://colab.research.google.com/github/deepmind/alphafold/blob/main/notebooks/AlphaFold.ipynb). The predicted structures were visualized and analyzed with UCSF Chimera version 1.14 (https://www.rbvi.ucsf.edu/chimera) (Pettersen et al., 2004).

## Supplementary Material

10.1242/joces.260288_sup1 Supplementary information
