# Supplementary material for: mNG-tagged fusion proteins and nanobodies to visualize tropomyosins in yeast and mammalian cells
Source: J Cell Sci. 2022 Sep 23;135(18):jcs260288. doi: 10.1242/jcs.260288 (PMC9592052; doi:10.1242/jcs.260288)
Supplement: Supplementary information [file joces-135-260288-s1.pdf]

Supplemental figure 1

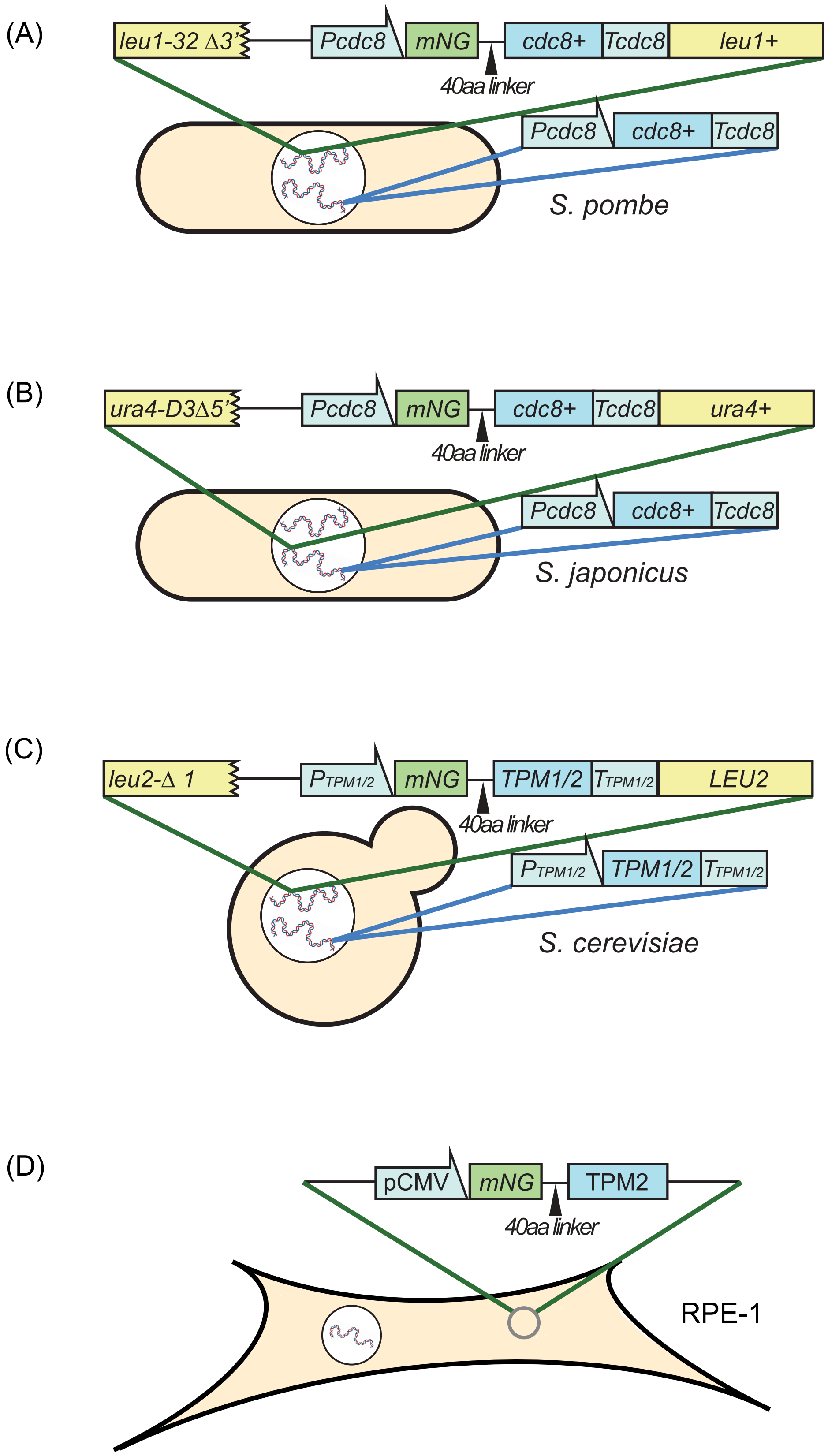

Journal of Cell Science • Supplementary information

Fig. S1. Strategy for tagging tropomyosin with mNeongreen: (A) Schematic illustration showing the insertion of P_{cdc8} mNG-40 amino acid linker-*cdc8* cassette at *leu1* locus. (B) Schematic illustration showing the insertion of P_{cdc8} mNG-40 amino acid linker-*cdc8* cassette at the *ura4* locus. The tag was expressed under the native *cdc8* promoter. (C) Schematic illustration showing the insertion of $P_{Tpm1/2}$ mNG-40 amino acid linker-*Tpm1/2* cassette at the *leu2* locus. (D) Schematic illustration showing the expression of mNG-40 aa linker-TPM2 under the control of a CMV promoter in an RPE-1 cell.

Journal of Cell Science • Supplementary information

Supplemental figure 2

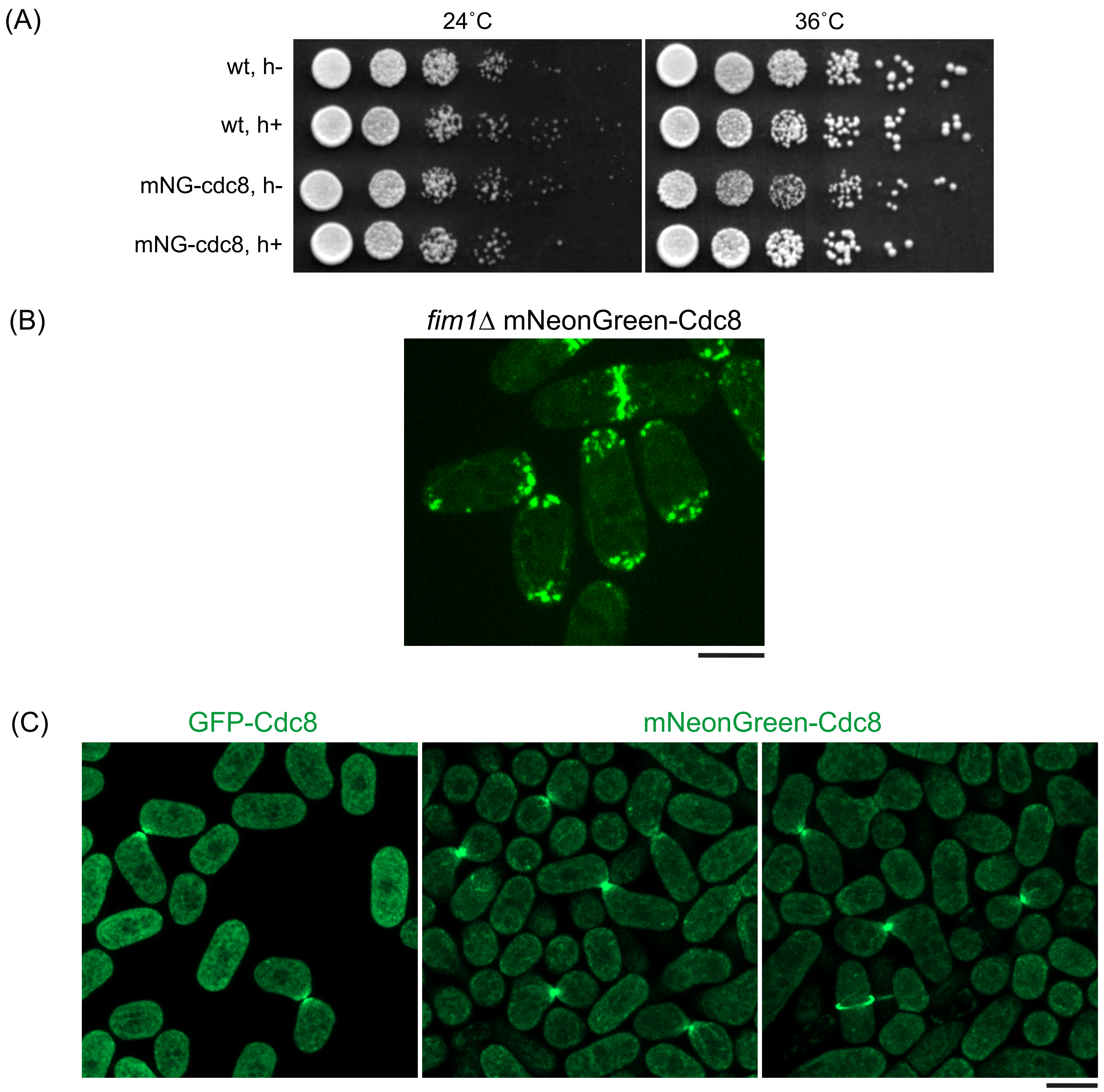

Fig. S2. Tagging Cdc8 with mNeonGreen in *S. pombe*. (A) Growth of strains was assessed by manual spot test. Untagged wild-type strains act as a control for mNG-Cdc8 strains. Cells were cultured in YEA media at 24˚C to saturation, subjected to six-fold serial dilution, and spotted onto YEA agar. Plates were incubated at 24˚C and 36˚C for 3 days before being photographed. (B) Fimbrin null mutant cells (*fim1*Δ) cells show obvious mNG-Cdc8 patches. (n=34). (C) Airyscan2 images of mating cells expressing GFP-Cdc8 (left) or mNG-Cdc8 (centre; right) which labels the fusion focus. mNG-Cdc8 allows detection of actin cables not visible with GFP-Cdc8 (n=4). Higher cytosolic GFP signals were detected in the GFP-cdc8 cells. Scale bars are 5μm.

Journal of Cell Science • Supplementary information

Supplemental figure 3

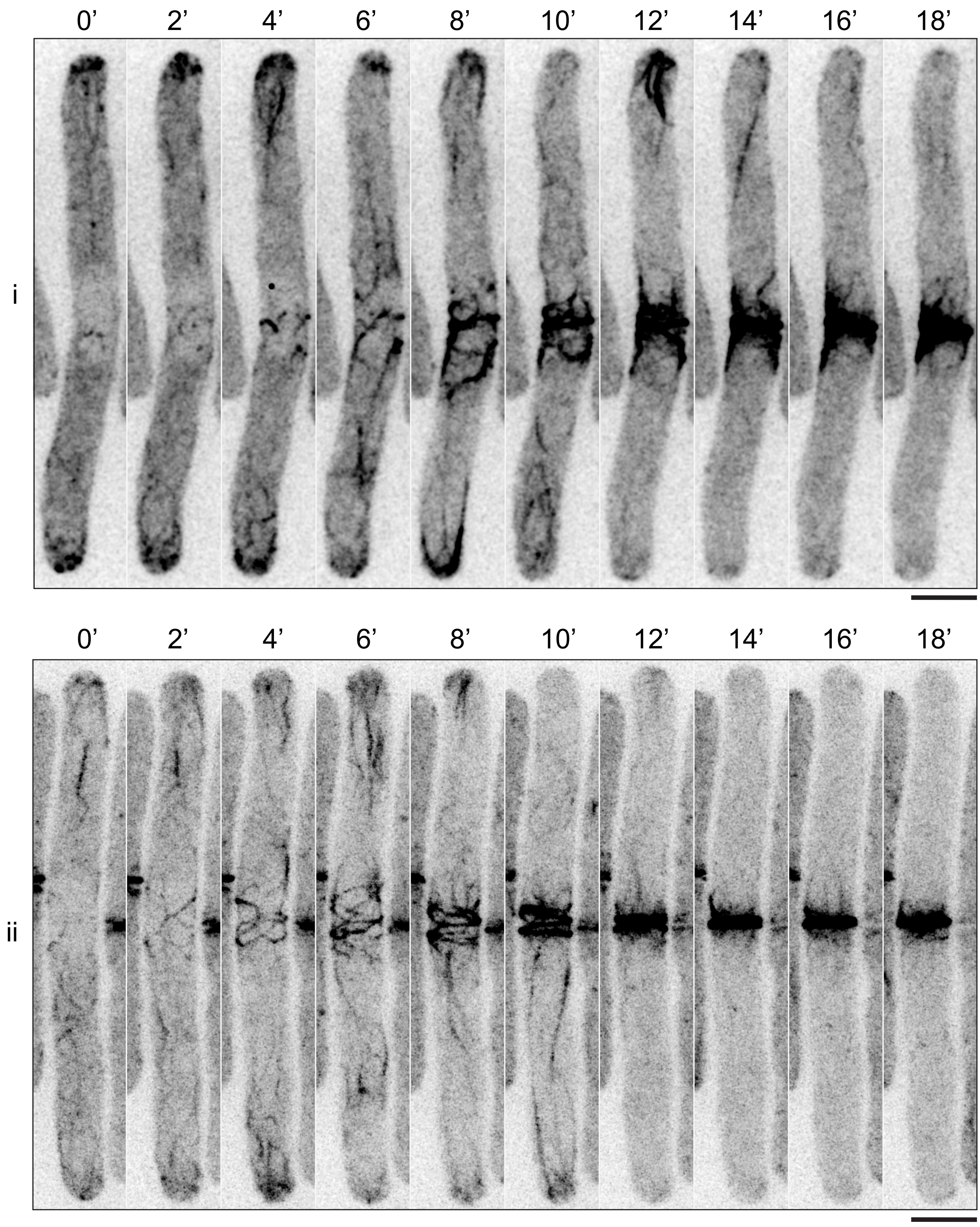

Fig. S3. Actomyosin ring assembly visualized using mNG-Cdc8 in elongated *cdc25*-22 cells. Panel i and ii are time-lapse images of two *S. pombe cdc25*-22 cells expressing mNG-Cdc8 demonstrating medial assembly of Cdc8 cables as well as flow of non-medial cables containing Cdc8-tropomyosin into the CAR. (n=65). Scale bars are 5µm.

Supplemental figure 4

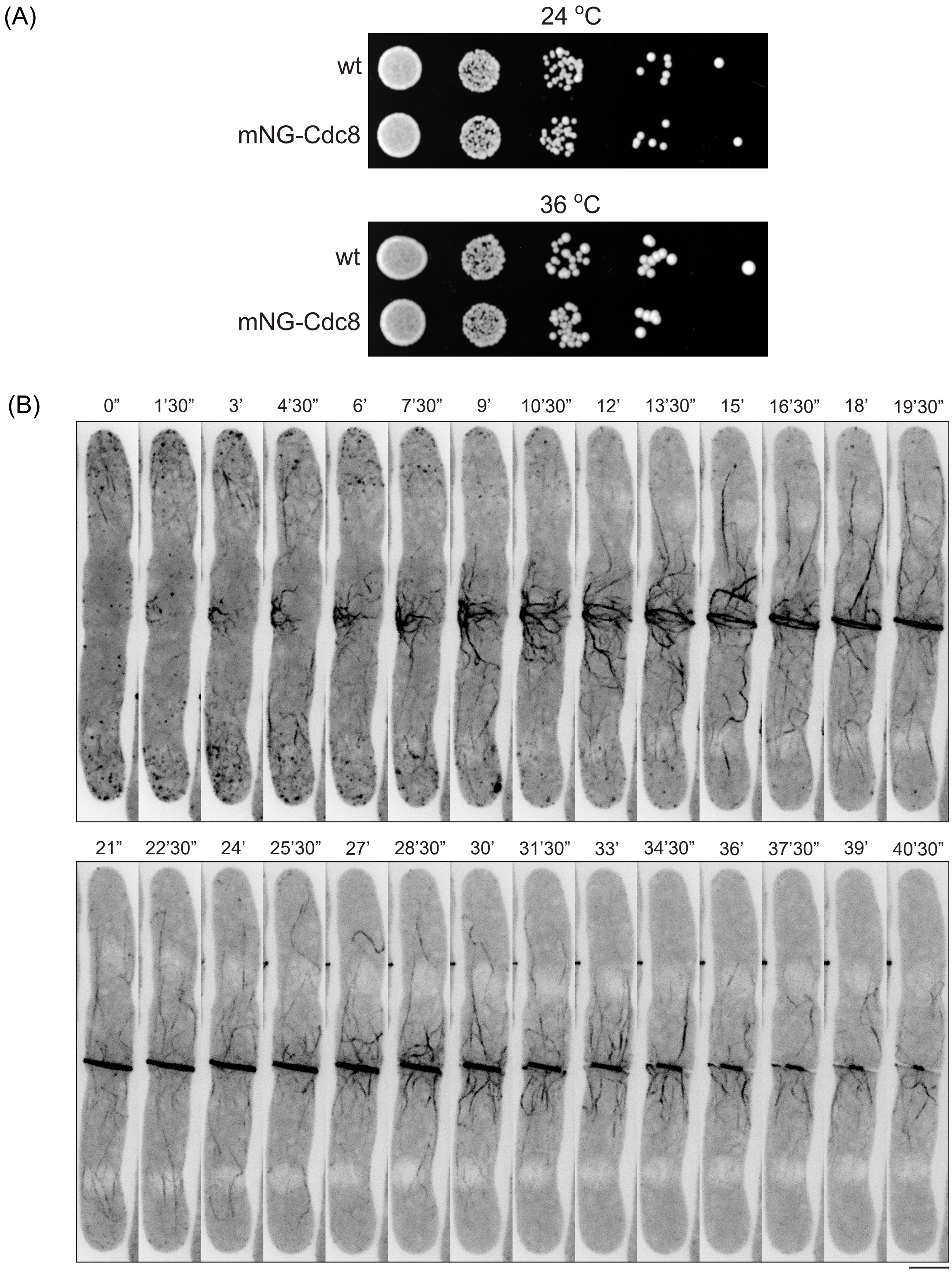

Fig. S4. Tagging Cdc8 with mNG in *S. japonicus*. (A) Growth of strains was assessed by manual spot test. Untagged wild-type strains act as a control for mNG-Cdc8 expressing strains. Cells were cultured in YEA media at 24˚C to saturation, subjected to five-fold serial dilution, and spotted onto YEA agar. Plates were incubated at 24˚C and 36˚C for 3 days before being photographed. (B) Time-lapse images of an *S. japonicus cdc25*-D9 cell expressing mNG-Cdc8 demonstrating medial assembly of Cdc8 cables as well as flow of non-medial cables containing Cdc8-tropomyosin into the CAR (n=12). Scale bar is 5μm.

Journal of Cell Science • Supplementary information

Supplemental figure 5

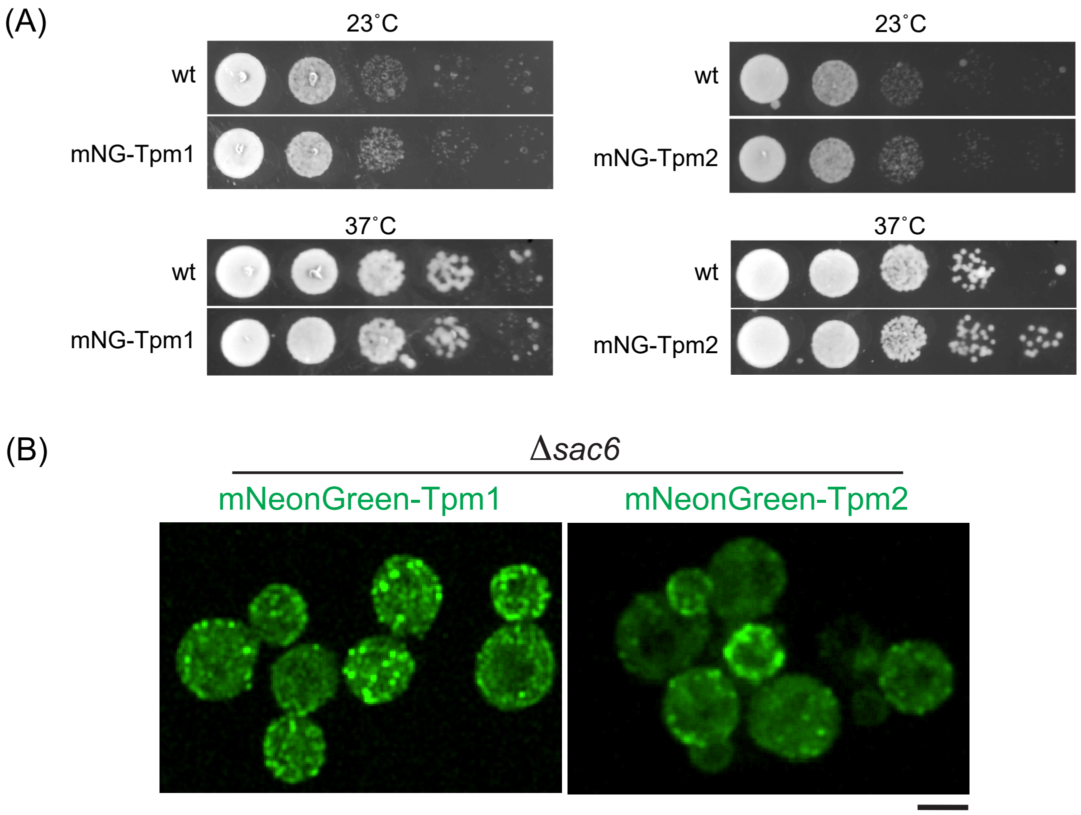

Fig. S5. Tagging Tpm1 and Tpm2 with mNG in *S. cerevisiae* cells. (A) Growth of strains was assessed by manual spot test. Untagged wild-type strains act as a control for mNG-tagged Tpm1 and Tpm2 strains. Cells were cultured in YPD media at 24˚C to saturation, subjected to five-fold serial dilution, and spotted onto YPD agar. Plates were incubated at 23˚C and 37˚C for 3 days before being photographed. (B) Fimbrin null mutant cells (*sac6*Δ cells) show obvious mNG-Tpm1 (n=209) and mNG-Tpm2 (114) patches. Scale bar is 5µm.

Journal of Cell Science • Supplementary information

Supplemental figure 6

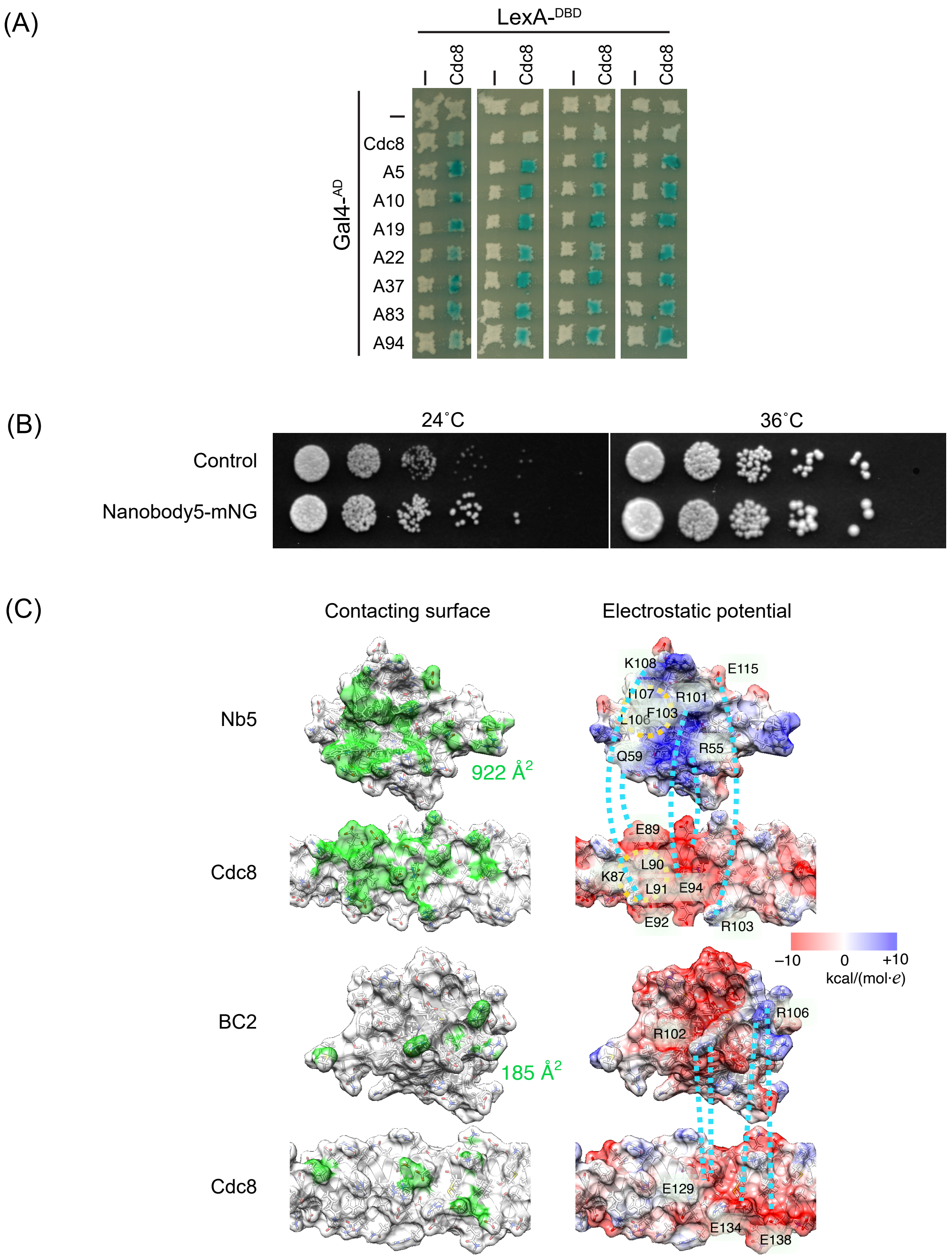

Journal of Cell Science • Supplementary information

Fig. S6. Cdc8 specific Nanobody-mNeonGreen in *S. pombe*. (A) Yeast two hybrid interaction shown between Sp Cdc8 and 7 camelid nanobodies. (B) Growth of strains was assessed by manual spot test. Untagged wild-type strains act as a control for Nb5-mNG strains. Cells were cultured in YEA media at 24˚C to saturation, subjected to six-fold serial dilution, and spotted onto YEA agar. Plates were incubated at 24˚C and 36˚C for 3 days before being photographed. (C) Alphafold 2 prediction of Cdc8-tropomyosin–Nb5 contacting surface sites and their electrostatic potential.

Journal of Cell Science • Supplementary information

Table S1. Yeast strains used in this work

	Strain number	Genotype
S. pombe	MBY101	*ade6*-210 *ura4*-D18 *leu1*-32 h-
	MBY102	*ade6*-210 *ura4*-D18 *leu1*-32 h+
	MBY192	*ura4*-D18 *leu1*-32 h-
	MBY12825	*leu1*-32 *pDUAL:p*^{cdc8}*:*mNeongreen-40aa:*cdc8 ura4*-D18 *ade6-210, cdc8+* h-
	MBY12828	*leu1*-32 *pDUAL:p*^{cdc8}*:*mNeongreen-40aa:*cdc8 ura4*-D18 *ade6*-210, *cdc8+* h+
	MBY12947	*leu1*-32 *pDUAL-p*^{cdc8}*:*mNeongreen-40aa:*cdc8 lys1<<pLYS1U-P*^{act1}*:lifeact*-mCherry:*ura4+ ade6*-210, *cdc8+*
	MBY12994	*fim1*Δ *leu1*-32 *pDUAL:p*^{cdc8}:mNeongreen-40aa:*cdc8, ura4*-D18, *ade6*-210, *cdc8+*
	MBY13071	*cdc25*-22 *leu1*-32 *pDUAL:p*^{cdc8}:mNeongreen-40aa:*cdc8 rlc1*-mCherry:*ura4+ ade6*-210 h+
	MBY13185	*leu1*-32 *p*^{adh11}:Nanobody5-mNeonGreen
	YSM3935	h90 *myo52*-tdTomato:*natMX leu1*-32:p^{cdc8}:mNeonGreen-*cdc8*:term^{cdc8}:term^{ADH1}:*leu1+* lys3+:p^{map3}:mCherry:term^{ADH1}:*bsdMX ade6*-M216 *ura4*-D18
	YSM3936	h90 *myo52*-tdTomato:*natMX leu1*-32:p^{ADH1}:*cdc8Nb5*-mNeonGreen:term^{ADH1}:*leu1+ ade6*-M210 *ura4*-294
	YSM3316	h90 *myo52*-tdTomato:*natMX leu1*-32:p^{ADH1}:*cdc8Nb5*-mNeonGreen:term^{ADH1}:*leu1+ ade6*-M210 *ura4*-294
S. japonicus	SOJ5	matsj-P2028 h-
	SOJ4909	*pcdc8*-mNeonGreen-40 a.a. linker-cdc8^{ORF}-*cdc8*$^{3'UTR}$::ura4+::ura4sj-D3 h+
	SOJ5001	*pcdc8*-mNeonGreen-40 a.a. linker-cdc8^{ORF}-*cdc8*$^{3'UTR}$::ura4+::ura4sj-D3 cdc25-D9:kanR:ura4+
	SOJ5221	*pcdc8*-mNeonGreen-40 a.a. linker-cdc8^{ORF}-*cdc8*$^{3'UTR}$::ura4+::ura4sj-D3 h-
S. cerevisiae	YSP002	ESM356 MATa *ura3*-52 *leu2*Δ1 *trp1*Δ63 *his3*Δ200 (wild type)
	YSP107	As YSP002 except pRS305-P$_{tpm1}$-mNG-40aaL-*tpm1*-T$_{tpm1}$-*leu2*
	YSP108	As YSP002 except pRS305-P$_{tpm2}$-mNG-40aaL-*tpm2*-T$_{tpm2}$-*leu2*
	YSP191	As YSP002 except pRS305-P$_{tpm1}$-mNG-40aaL-*tpm1*-T$_{tpm1}$-*leu2* Δ*sac6::His3MX6 abp1*-tdtomato::*natNT2*
	YSP192	As YSP002 except pRS305-P$_{tpm2}$-mNG-40aaL-*tpm2*-T$_{tpm2}$-*leu2* Δ*sac6::His3MX6 abp1*-tdtomato::*natNT2*

Table S2. Plasmids used in this study

Plasmid name	Description	Reference			
**pTH8-77**	pDUAL-cdc8(5'UTR)-mNG-40 a.a. linker-cdc8-cdc8(3'UTR)	This Study			
**pMB117**	pDUAL-Padh11-Nb A5-40 a.a. linker-mNG	This Study			
**pSO1233**	pBluescriptSK(+)-ura4+(S. japonicus)-cdc8(5'UTR)-mNG-40 a.a. linker-cdc8-cdc8(3'UTR)	This Study			
**piSP347**	pRS305-Ptpm1-mNG-40aaL-tpm1-Ttpm1	This Study			
**piSP349**	pRS305-Ptpm2-mNG-40aaL-tpm2-Ttpm2	This Study			
**piSP23**	pFA6a-His3MX6	Knop et al 1999			
**pMB128**	pDUAL-Padh11-mNeongreen-40aaL	MB lab			
**piSP112**	pMM5s	This Study			
**piSP113**	pMM6s	This Study			
**piSP600**	pMM5S-Cdc8	This Study			
**piSP604**	pMM6S-Cdc8	This Study			
**piSP198**	pMM5S-Cdc8 Nb A5	This Study			
**piSP200**	pMM5S-Cdc8 Nb A10	This Study			
**piSP202**	pMM5S-Cdc8 Nb A19	This Study			
**piSP203**	pMM5S-Cdc8 Nb A22	This Study			
**piSP204**	pMM5S-Cdc8 Nb A37	This Study			
**piSP206**	pMM5S-Cdc8 Nb A83	This Study			
**piSP207**	pMM5S-Cdc8 Nb A94	This Study			
**piSP209**	pMM6S-Cdc8 Nb A5	This Study			
**piSP211**	pMM6S-Cdc8 Nb A10	This Study			
**piSP213**	pMM6S-Cdc8 Nb A19	This Study			
**piSP215**	pMM6S-Cdc8 Nb A22	This Study			
**piSP217**	pMM6S-Cdc8 Nb A37	This Study			
**piSP218**	pMM6S-Cdc8 Nb A83	This Study			
**piSP219**	pMM6S-Cdc8 Nb A94	This Study			

Journal of Cell Science • Supplementary information

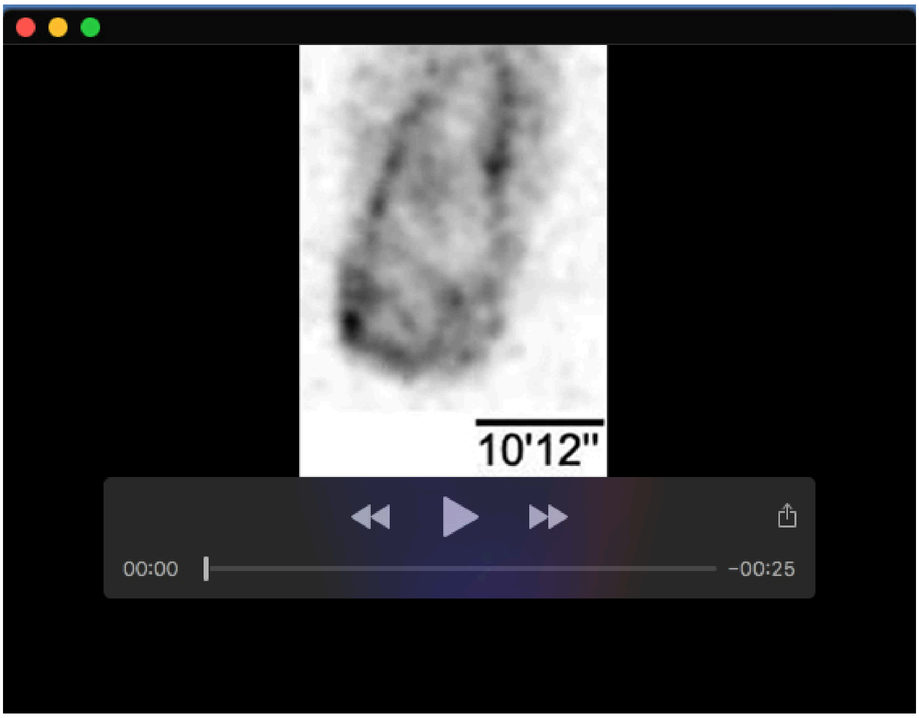

Movie 1. *S. pombe* mNG-Cdc8 patch and cable dynamics.

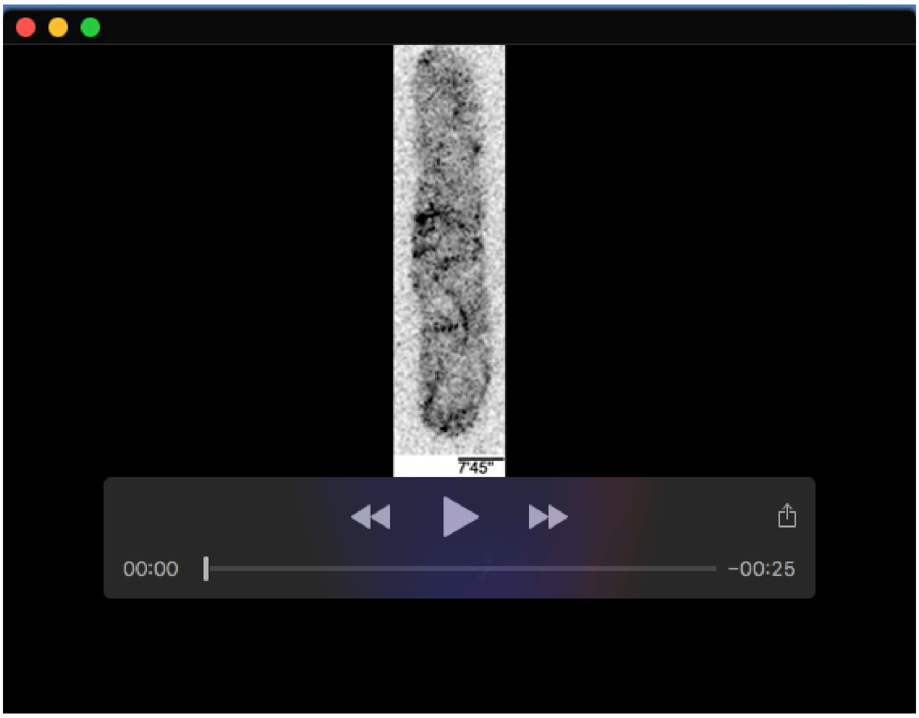

Movie 2. mNG-Cdc8 dynamics during CAR assembly in *S. pombe*

Journal of Cell Science • Supplementary information

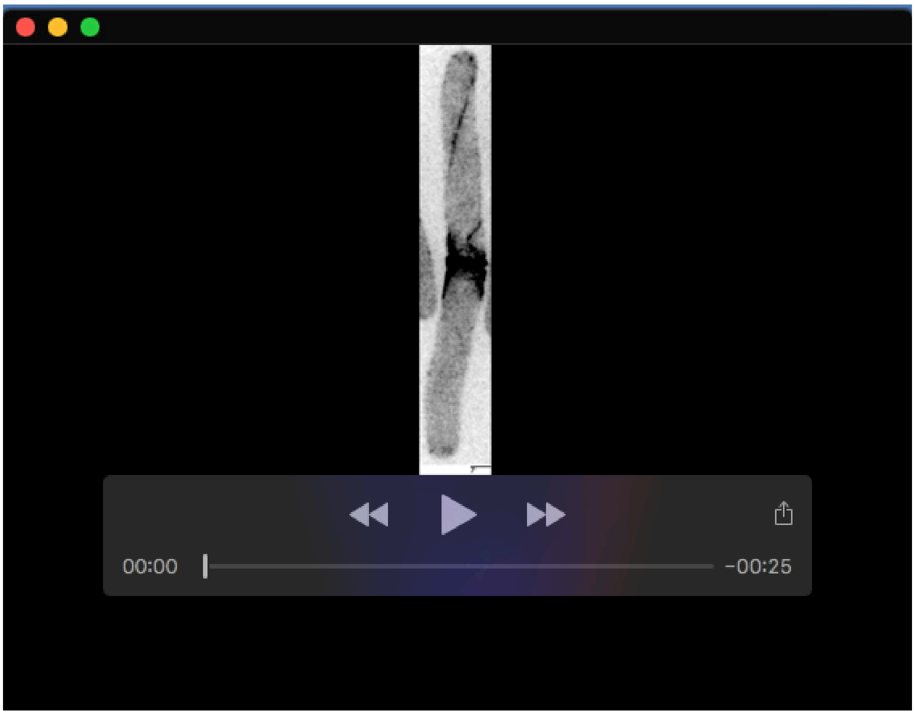

Movie 3. mNG-Cdc8 dynamics during CAR assembly in highly elongated *S. pombe cdc25*- 22 cells, demonstrating flow of Cdc8 cables into the CAR during its assembly.

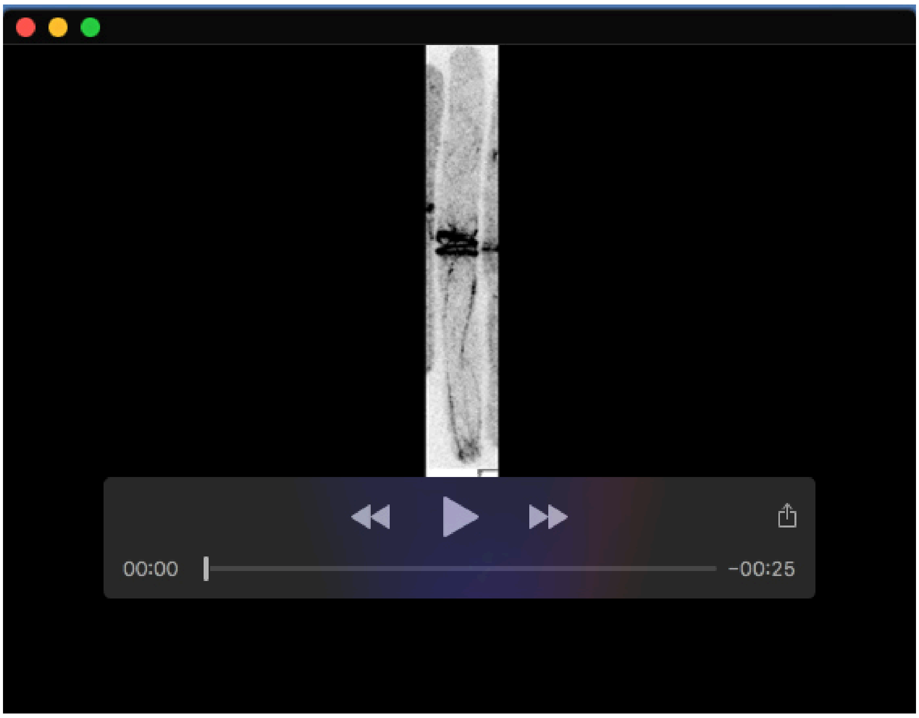

Movie 4. mNG-Cdc8 dynamics during CAR assembly in highly elongated *S. pombe cdc25*- 22 cells, demonstrating flow of Cdc8 cables into the CAR during its assembly.

Journal of Cell Science • Supplementary information

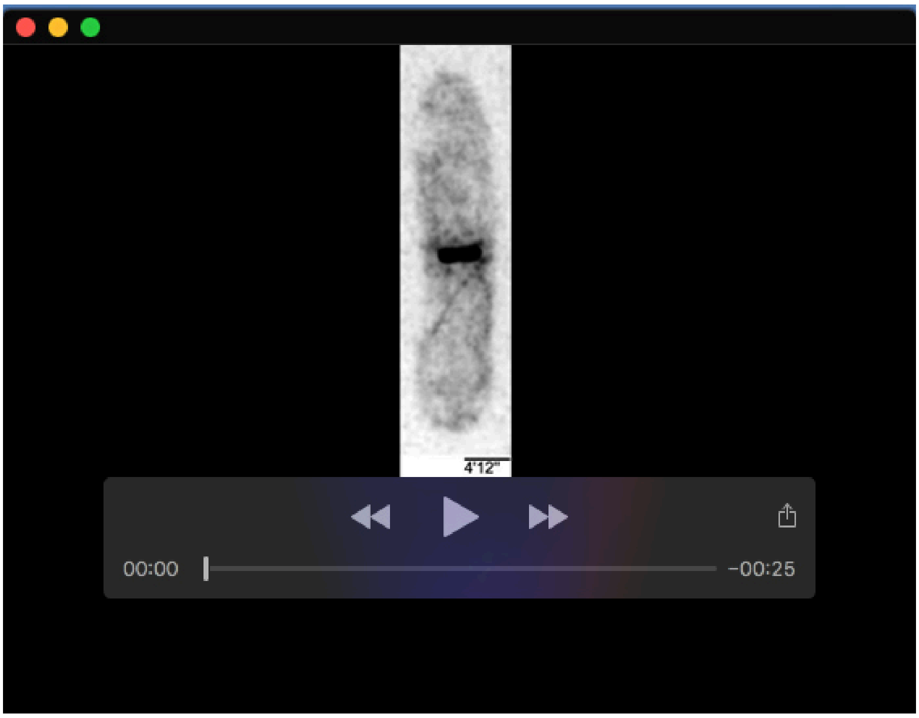

Movie 5. mNG-Cdc8 dynamics and cable expulsion during CAR constriction in *S. pombe*.

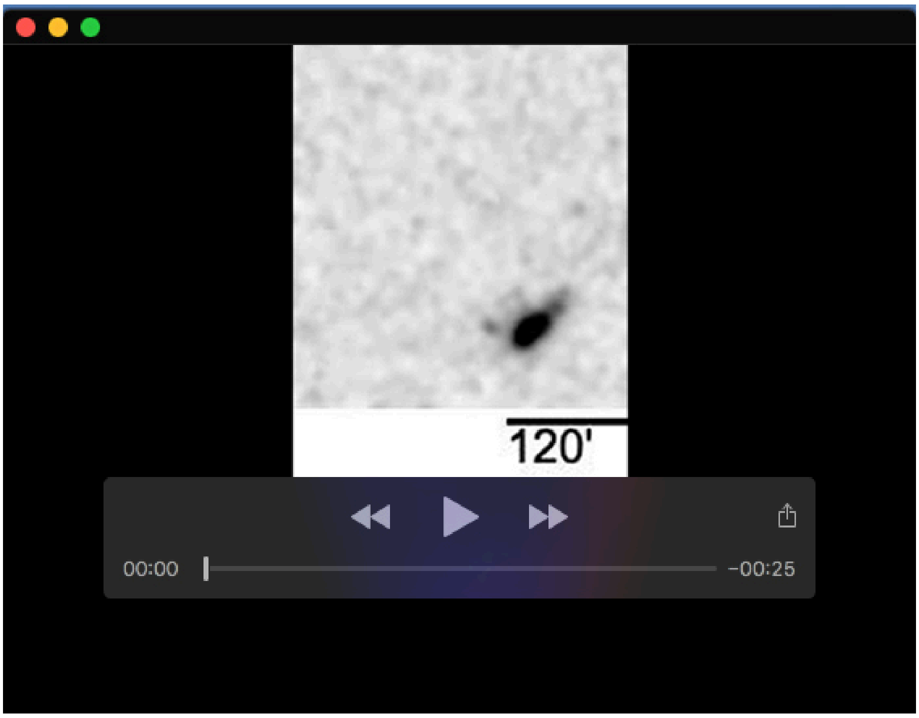

Movie 6. Dynamics of mNG-Cdc8 during ATP-dependent constriction of CARs within *S. pombe* cell ghosts.

Journal of Cell Science • Supplementary information

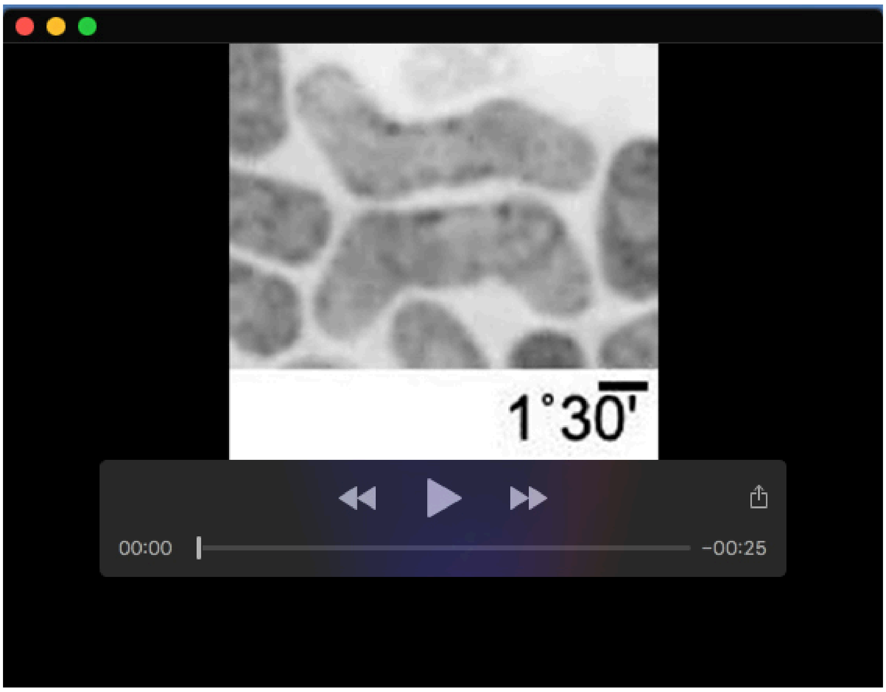

Movie 7. Dynamics of *S. pombe* mNG-Cdc8 during mating and sporulation.

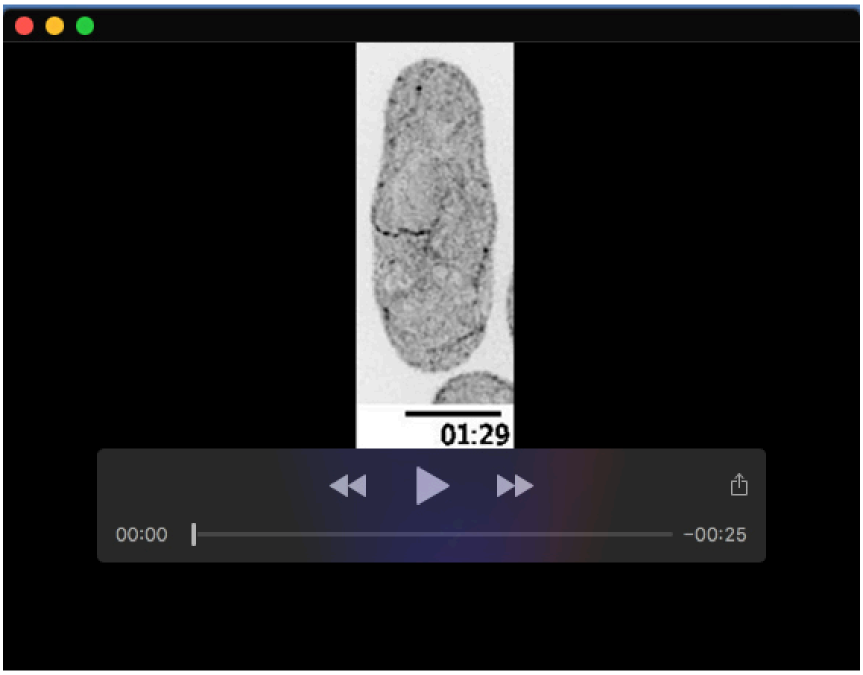

Movie 8. Dynamics of *S. japonicus* mNG-Cdc8 during CAR assembly and constriction.

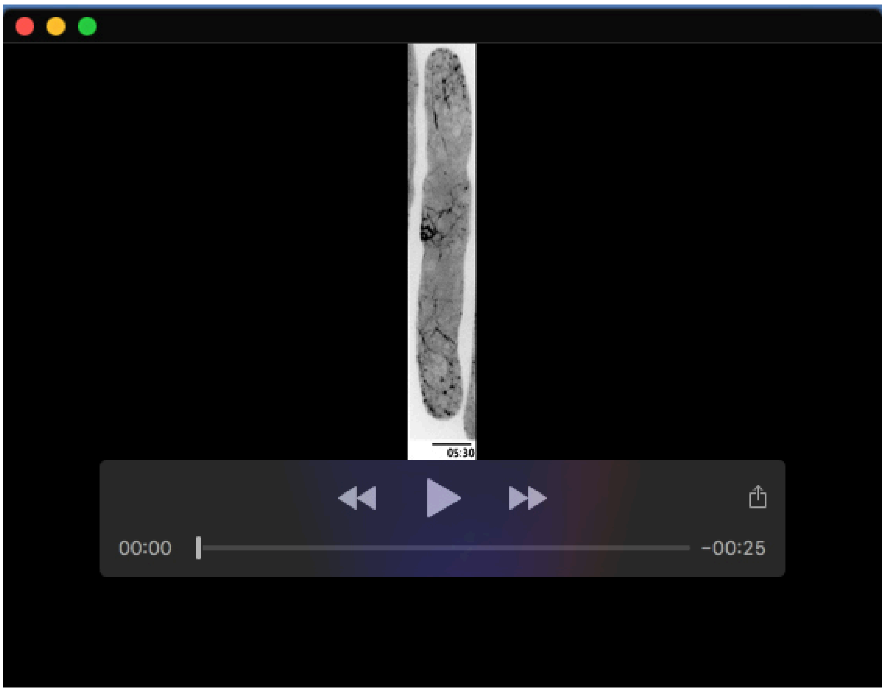

Movie 9. Dynamics of *S. japonicus* mNG-Cdc8 during CAR assembly and constriction in elongated *cdc25*-D9 cells.

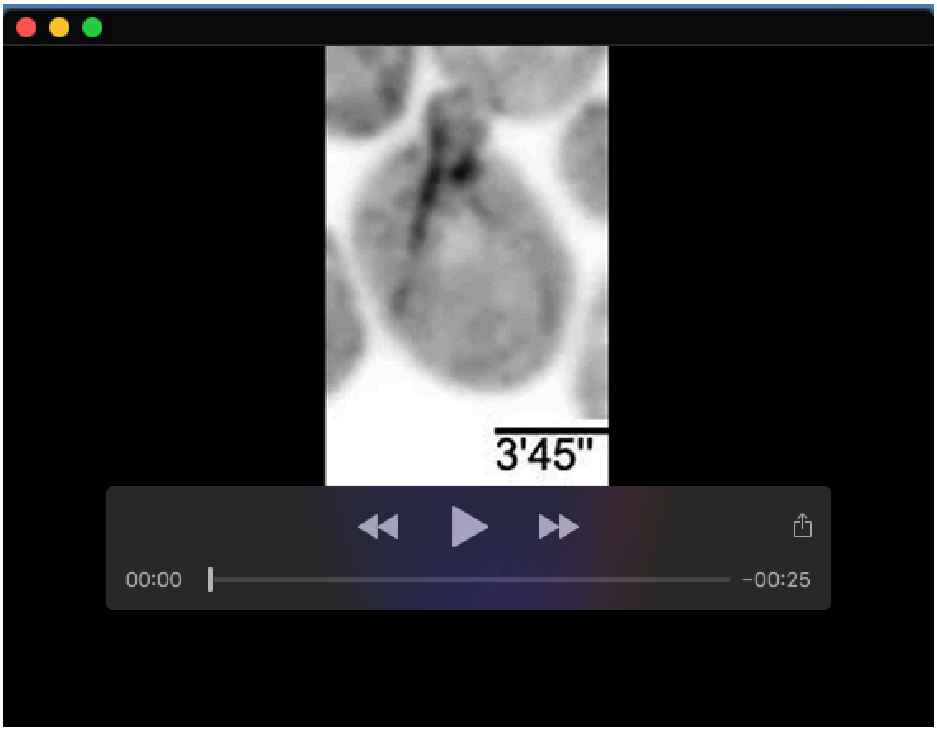

Movie 10. Dynamics of *S. cerevisiae* mNG-Tpm1 in cables.

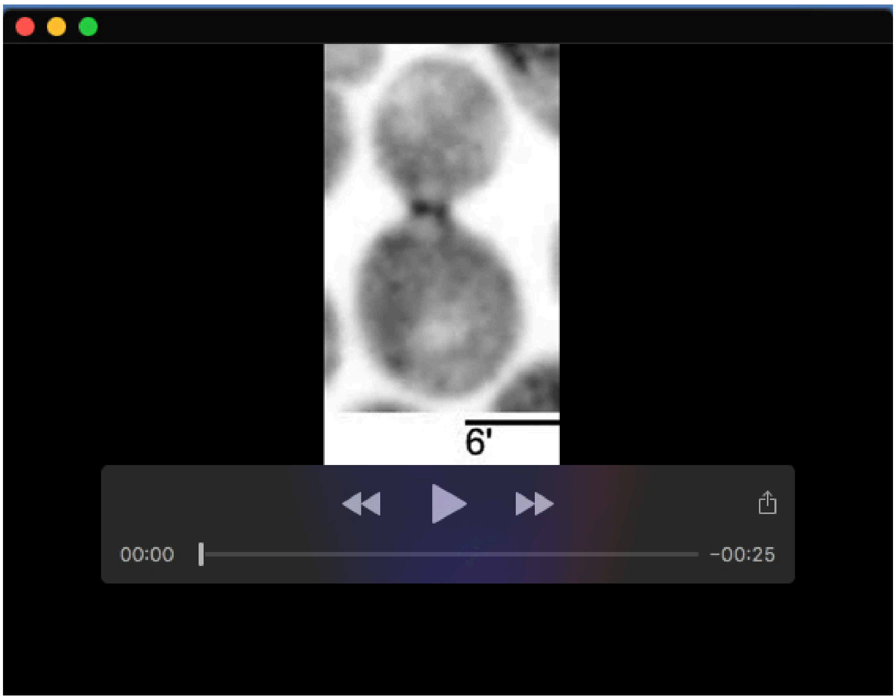

Movie 11. Dynamics of *S. cerevisiae* mNG-Tpm1 in the CAR.

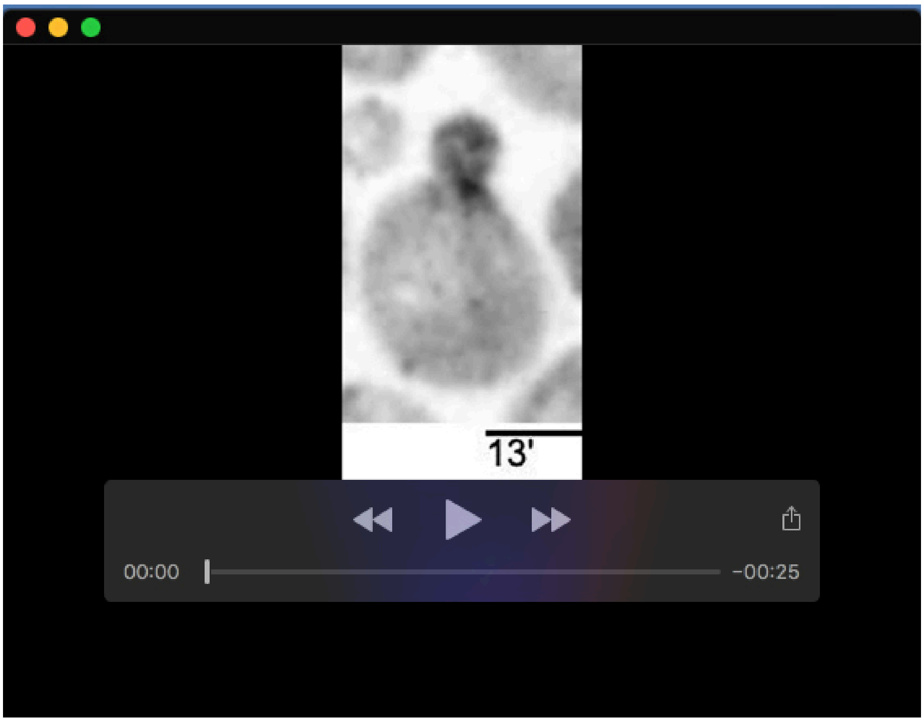

Movie 12. Dynamics of *S. cerevisiae* mNG-Tpm2 in cables.

Journal of Cell Science • Supplementary information

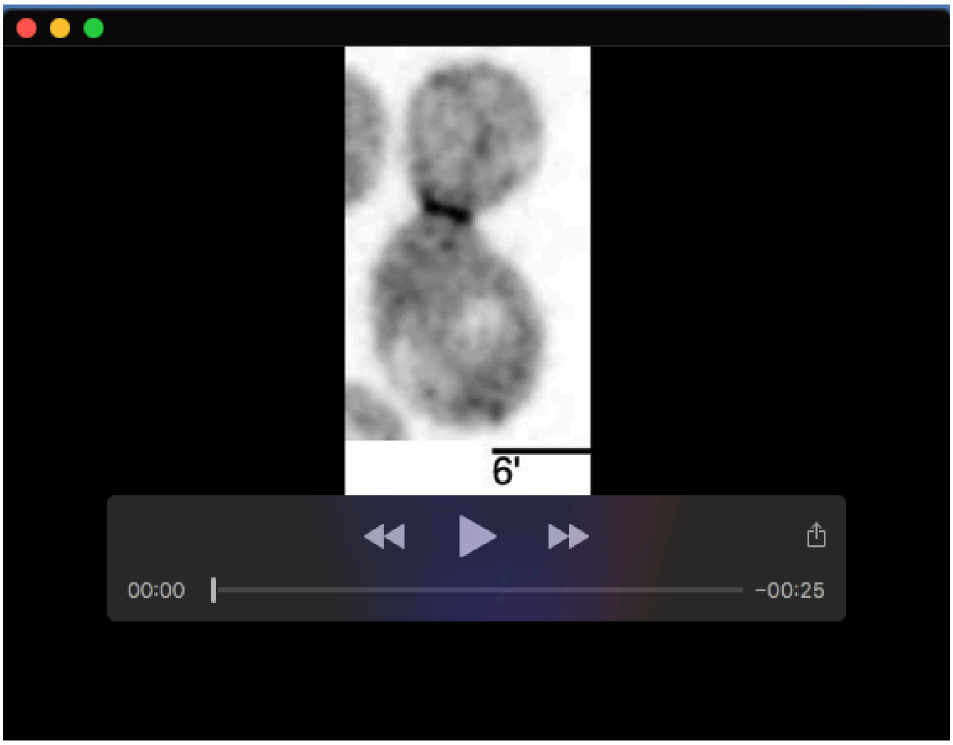

Movie 13. Dynamics of *S. cerevisiae* mNG-Tpm2 in the CAR.

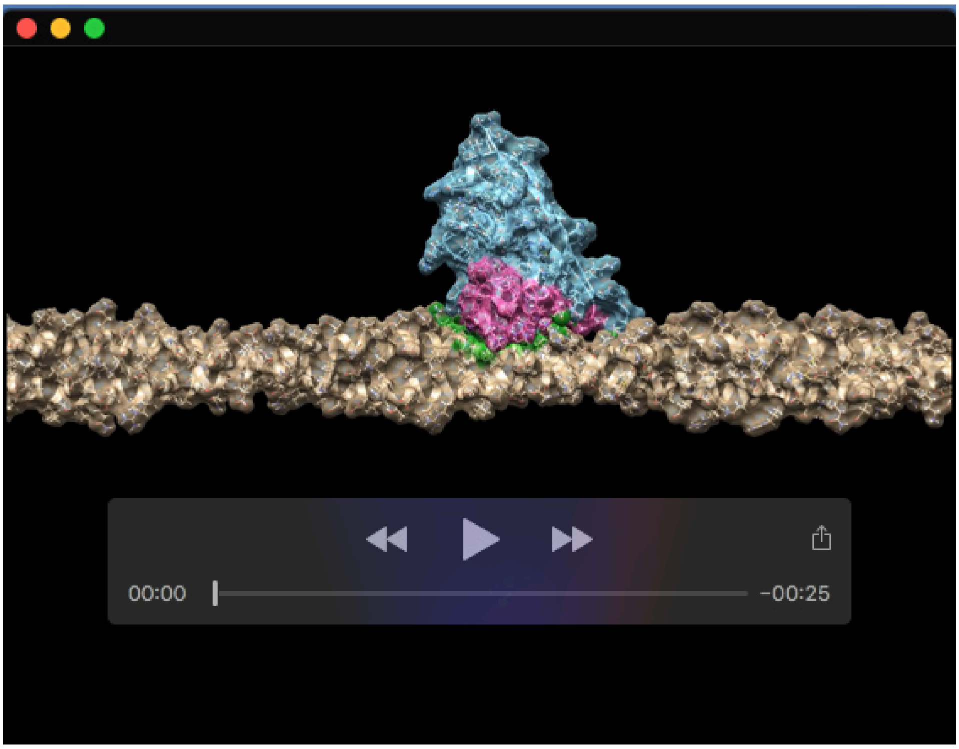

Movie 14. Simulation of Cdc8-Nanobody 5 interaction with Cdc8 dimer.

Journal of Cell Science • Supplementary information

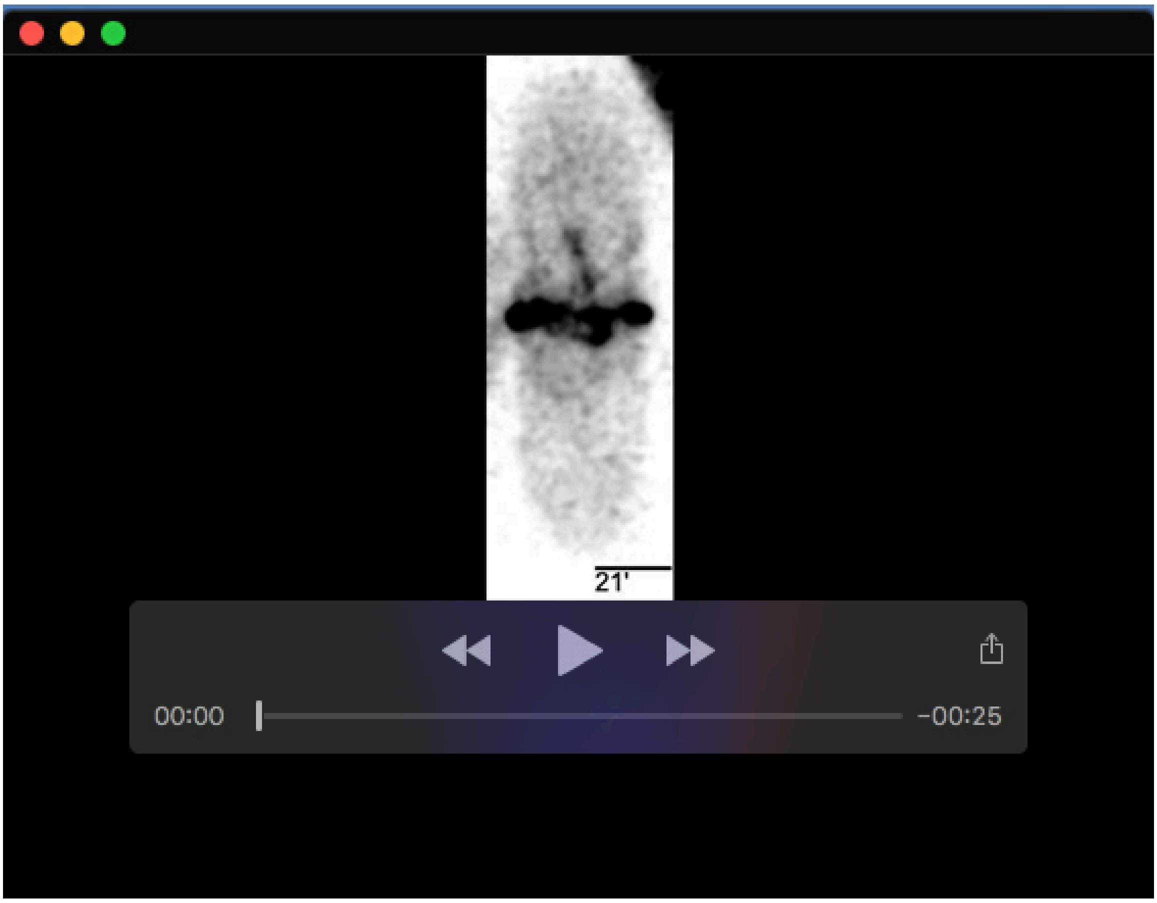

Movie 15. Dynamics of mNG-Nb5 during CAR assembly in *S. pombe*.
