# Supplementary material for: mNG-tagged fusion proteins and nanobodies to visualize tropomyosins in yeast and mammalian cells
Source: J Cell Sci. 2022 Sep 23;135(18):jcs260288. doi: 10.1242/jcs.260288 (PMC9592052; doi:10.1242/jcs.260288)
Supplement: Supplementary file 1 [file joces-135-260288_review_history.pdf]

Journal of Cell Science

mNG-tagged fusion proteins and nanobodies to visualize tropomyosins in yeast and mammalian cells

Tomoyuki Hatano, Tzer Chyn Lim, Ingrid Billault-Chaumartin, Anubhav Dhar, Ying Gu, Teresa Massam-Wu, William Scott, Sushmitha Adishesha, Bernardo Chapa-y-Lazo, Luke Springall, Lavanya Sivashanmugam, Masanori Mishima, Sophie G. Martin, Snezhana Oliferenko, Saravanan Palani and Mohan K. Balasubramanian
DOI: 10.1242/jcs.260288

Editor: Michael Way

Review timeline

Original submission:	27 May 2022
Editorial decision:	27 June 2022
First revision received:	12 August 2022
Accepted:	15 August 2022

Original submission

First decision letter

MS ID#: JOCES/2022/260288

MS TITLE: mNeonGreen-tagged fusion proteins and nanobodies reveal localization of tropomyosin to patches, cables, and contractile actomyosin rings in live yeast cells

AUTHORS: Tomoyuki Hatano, Tzer Chyn Lim, Ingrid Billault-Chaumartin, Anubhav Dhar, Ying Gu, Teresa Massam-Wu, Sushmitha Adishesha, Luke Springall, William Scott, Lavanya Sivashanmugam, Masanori Mishima, Sophie G Martin, Snezhana Oliferenko, Saravanan Palani, and MOHAN K BALASUBRAMANIAN
ARTICLE TYPE: Tools and Resources

We have now reached a decision on the above manuscript.

To see the reviewers' reports and a copy of this decision letter, please go to: https://submit-jcs.biologists.organd click on the 'Manuscripts with Decisions' queue in the Author Area. (Corresponding author only has access to reviews.)

As you will see, the reviewers all gave favourable reports but raised some points that will require amendments to your manuscript. In particular I think it is important to provide some quantification as suggested by reviewer 3. I also think that you should try and come up with a more succinct title that encapsulates the main message of the paper rather than all the actin structures your probe labels. I hope that you will be able to carry these out because I would like to be able to accept your paper.

Please ensure that you clearly highlight all changes made in the revised manuscript. Please avoid using 'Tracked changes' in Word files as these are lost in PDF conversion.

I should be grateful if you would also provide a point-by-point response detailing how you have dealt with the points raised by the reviewers in the 'Response to Reviewers' box. Please attend to all of the reviewers' comments. If you do not agree with any of their criticisms or suggestions please explain clearly why this is so.

© 2022. Published by The Company of Biologists under the terms of the Creative Commons Attribution License (https://creativecommons.org/licenses/by/4.0/).

Reviewer 1

Advance summary and potential significance to field

Tropomyosins are essential actin binding proteins that confer stability on actin filaments. To date, they have been very difficult to study in live cells as reagents have not had sufficiently strong signals to detect in some actin structures or for long periods. This work presents a signficant advance in this area and reports on the generation of tools that permits real time imaging of tropomyposins in living cells. The study has primarily focused on analysis of the tropomyosin Cdc8 in S.pombe cells but then demonstrates proof of principle in two other yeasts (S.japonicus and S.cerevisiae) and also in mammalian RPE cells. The manuscript is submitted as a tools and techniques paper and as such the data provided are strong evidence for the efficacy of both the mNG(long linker) tagging and the Nanobody approaches to future studies on tropomyosin function. Analysis of the function of the various mammalian tropomyosin isoforms would be a particular area that would likely welcome further application of these methods to address long outstanding questions in the field.

Comments for the author

I would recommend minor revisions, largely to ensure the widest viewing and possible adoption of the approaches.
Most important would be to include the figures of the mammalian RPE expression in the main paper(rather than people having to find this part in supplementary). A control should be included of cells prior to transient expression of the construct as this would indicate whether the expression itself alters the actin cytoskeleton.
The title could be amended to reflect the idea that the article is about tools but could also mention mammalian cells. If this isn't in the title I feel that some people will think that it is yeast specific approaches and might not look further.

Reviewer 2

Advance summary and potential significance to field

Hatano T. et al. reported a new imaging toolset to investigate living cell tropomyosin, which is a long-missing biomarker for live-cell imaging for different mechanisms of actin cytoskeleton assembly and involved cellular processes.
Tropomyosin is a conserved F-actin binding protein that cooperates with other F-actin binding proteins during the treadmilling process. Due to the long coiled-coil structures, getting a functional fluorescent protein-tagged version for imaging its filamentous structures in living cells has been challenging. In addition to the reported reporter, Although the biochemical competition between tropomyosin and actin-binding and bundling proteins has been reported, living cell imaging during complex cellular conditions was not possible. Tropomyosin localization on patch structure in fimbrin mutants is an excellent demonstration to study different F-actin binding proteins and their cooperation with tropomyosin. Interestingly although fimbrin directly competes with tropomyosin in vitro via biochemical assay, they have distinct primary localizations in vivo, on actin patch and actin cable, respectively. Without Sac6, it seems tropomyosin starts to occupy patch sites.

Here a mNeon-Green version of different tropomyosin homologs showed filamentous structures that are suitable for time-lapse imaging in real-time. The filaments highly colocalize with well-used actin markers, such as LifeAct-mCherry. mNG-Cdc8 and mNG-Tpm1/2 showed as valuable biomarkers of tropomyosin in S. japonicus, S. cerevisiae, and human RPE cells. These successful applications in different eukaryotic species have also proved their universality to inspire a border application in the future.

Comments for the author

The study is well designed and performed. The tropomyosin imaging tools have broad applications in cell biology. I only have a few minor comments.

© 2022. Published by The Company of Biologists under the terms of the Creative Commons Attribution License (https://creativecommons.org/licenses/by/4.0/).

1. This story has also efficiently developed several nanobodies against different regions of tropomyosin. Besides the potential binding mode between Nb and antigen using Alphafold, may the author list the sequence of the corresponding tropomyosin fragments that have been used to generate these nanobodies or at least the Nb5 that is reported in this manuscript?
2. Please indicate the ecotype background of yeast strains in the method.
3. Please provide a list of plasmids that were used to generate yeast strains as supplementary files, which will be very useful for the community.

Reviewer 3

Advance summary and potential significance to field

Tropomyosin labeling has proven difficult to achieve, especially in live cells. Current strategies are limited to low expression of GFP-tagged proteins, which only works in some species and does not label certain actin networks in the cell where tropomyosin naturally binds.
This manuscript reports two novel labeling strategies that may work more generally. The first strategy is an N-terminal labeling strategy optimized with mNeonGreen and a 40 aa long linker, which here allows labeling of five different tropomyosins from four different species (3 yeasts and one mammalian cell line). This new marker is likely to become a reference, as it allows detection of tropomyosins more accurately. The second strategy is based on the use of nanobodies. This second strategy seems currently more of a proof of concept and still requires development efforts, but shows encouraging potential for the future.
In parallel, this work reports few original findings and nicely confirms previous observations - including, weak but detectable presence of tropomyosin on actin patches in yeast, beautiful patch migration along actin cables and flows of actin cables to the cytokinetic ring.

Comments for the author

1/ I have a very positive opinion about this work but regret the general lack of quantification on the current version of the manuscript. From the nice images and movies, it should be possible to have quantification of:
a/ relative intensity of tropomyosin to actin signals for each actin filament structure, in order to have a quantitative appreciation of tropomyosin preference for the different networks.
b/ patch velocities along actin cables.
2/ I could not find any information about the Nterminal state of the labeled tropomyosins. Do the authors know if tropomyosin acetylation is preserved? If not, would mimicking residues increase the affinity of tropomyosin and improve signals?
3/ I am not sure I understand the authors' conclusion about the locations of Tpm1 and Tpm2 in cerevisiae. Could the authors clearly state whether they can conclude on a different localization pattern of these two tropomyosins, or whether this apparent difference is rather due to imaging limitations?
4/ Supp Figure 4E gives the impression that not all stress fibers are equally decorated by TPM2. Is this true or a misleading impression?

First revision

Author response to reviewers' comments

Reviewer 1 Advance Summary and Potential Significance to Field:

Tropomyosins are essential actin binding proteins that confer stability on actin filaments. To date, they have been very difficult to study in live cells as reagents have not had sufficiently strong signals to detect in some actin structures or for long periods. This work presents a significant advance in this area and reports on the generation of tools that permits real time imaging of tropomyosins in living cells. The study has primarily focused on analysis of the tropomyosin Cdc8 in S.pombe cells but then demonstrates proof of principle in two other yeasts

© 2022. Published by The Company of Biologists under the terms of the Creative Commons Attribution License (https://creativecommons.org/licenses/by/4.0/).

(S.japonicus and S.cerevisiae) and also in mammalian RPE cells. The manuscript is submitted as a tools and techniques paper and as such the data provided are strong evidence for the efficacy of both the mNG (long linker) tagging and the Nanobody approaches to future studies on tropomyosin function. Analysis of the function of the various mammalian tropomyosin isoforms would be a particular area that would likely welcome further application of these methods to address long outstanding questions in the field.

Reviewer 1 Comments for the Author:

I would recommend minor revisions, largely to ensure the widest viewing and possible adoption of the approaches. Most important would be to include the figures of the mammalian RPE expression in the main paper(rather than people having to find this part in supplementary). A control should be included of cells prior to transient expression of the construct as this would indicate whether the expression itself alters the actin cytoskeleton.

Response: Done as suggested and included in Figure 5 in the main manuscript.

The title could be amended to reflect the idea that the article is about tools but could also mention mammalian cells. If this isn't in the title I feel that some people will think that it is yeast specific approaches and might not look further.

Response: Done as suggested.

Reviewer 2 Advance Summary and Potential Significance to Field:
Hatano T. et al. reported a new imaging toolset to investigate living cell tropomyosin, which is a long-missing biomarker for live-cell imaging for different mechanisms of actin cytoskeleton assembly and involved cellular processes. Tropomyosin is a conserved F-actin binding protein that cooperates with other F-actin binding proteins during the treadmilling process. Due to the long coiled-coil structures, getting a functional fluorescent protein-tagged version for imaging its filamentous structures in living cells has been challenging. In addition to the reported reporter, Although the biochemical competition between tropomyosin and actin-binding and bundling proteins has been reported, living cell imaging during complex cellular conditions was not possible. Tropomyosin localization on patch structure in fimbrin mutants is an excellent demonstration to study different F-actin binding proteins and their cooperation with tropomyosin. Interestingly although fimbrin directly competes with tropomyosin in vitro via biochemical assay, they have distinct primary localizations in vivo, on actin patch and actin cable, respectively. Without Sac6, it seems tropomyosin starts to occupy patch sites.

Here a mNeon-Green version of different tropomyosin homologs showed filamentous structures that are suitable for time-lapse imaging in real-time. The filaments highly colocalize with well-used actin markers, such as LifeAct-mCherry. mNG-Cdc8 and mNG-Tpm1/2 showed as valuable biomarkers of tropomyosin in S. japonicus, S. cerevisiae, and human RPE cells. These successful applications in different eukaryotic species have also proved their universality to inspire a border application in the future.

Reviewer 2 Comments for the Author:

The study is well designed and performed. The tropomyosin imaging tools have broad applications in cell biology. I only have a few minor comments.

1. This story has also efficiently developed several nanobodies against different regions of tropomyosin. Besides the potential binding mode between Nb and antigen using Alphafold, may the author list the sequence of the corresponding tropomyosin fragments that have been used to generate these nanobodies or at least the Nb5 that is reported in this manuscript?

Response: The entire Cdc8 tropomyosin gene was expressed in E. coli and purified protein used to screen for nanobodies. The sequence of Nb5 is now included in the materials and methods section.

© 2022. Published by The Company of Biologists under the terms of the Creative Commons Attribution License (https://creativecommons.org/licenses/by/4.0/).

2. Please indicate the ecotype background of yeast strains in the method.

Response: The *S. pombe* strains were all based on the 968 h^{90} background. The *S. japonicus* strains were based on NIG2021 and *S. cerevisiae* were based on the S288c. These are now mentioned in the methods.

3. Please provide a list of plasmids that were used to generate yeast strains as supplementary files, which will be very useful for the community.

Response: Provided as requested.

Reviewer 3 Advance Summary and Potential Significance to Field:
Tropomyosin labeling has proven difficult to achieve, especially in live cells. Current strategies are limited to low expression of GFP-tagged proteins, which only works in some species and does not label certain actin networks in the cell where tropomyosin naturally binds.

This manuscript reports two novel labeling strategies that may work more generally. The first strategy is an N-terminal labeling strategy optimized with mNeonGreen and a 40 aa long linker, which here allows labeling of five different tropomyosins from four different species (3 yeasts and one mammalian cell line). This new marker is likely to become a reference, as it allows detection of tropomyosins more accurately. The second strategy is based on the use of nanobodies. This second strategy seems currently more of a proof of concept and still requires development efforts but shows encouraging potential for the future.

In parallel, this work reports few original findings and nicely confirms previous observations - including, weak but detectable presence of tropomyosin on actin patches in yeast, beautiful patch migration along actin cables and flows of actin cables to the cytokinetic ring.

Reviewer 3 Comments for the Author:

1/ I have a very positive opinion about this work but regret the general lack of quantification on the current version of the manuscript. From the nice images and movies, it should be possible to have quantification of:

a/ relative intensity of tropomyosin to actin signals for each actin filament structure, in order to have a quantitative appreciation of tropomyosin preference for the different networks.

Response: The relative intensity of tropomyosin to actin in patches, cables, and rings have been quantified using a trainable weka segmentation strategy we developed. The data are now provided in the revised version of figure 1.

b/ patch velocities along actin cables.

Response: Although this is an interesting issue, the fact that patches are fainter compared to actin patches makes this more difficult. Furthermore, these analyses can be better performed with a strain expressing lifeact-GFP or mNG, which is not the main subject of the current work. As a result, we believe this analysis is beyond the scope of the current work and we will do this in a more elaborate work we carry out using these fluorescent tools.

2/ I could not find any information about the Nterminal state of the labeled tropomyosins. Do the authors know if tropomyosin acetylation is preserved? If not, would mimicking residues increase the affinity of tropomyosin and improve signals?

Response: This is an important and interesting point. We believe the tagged tropomyosins are mimic acetylated state and do not affect head to tail polymerization based on several past *in vitro* and *in vivo* studies with fission yeast and vertebrate tropomyosin fusions. We have introduced these references into the manuscript.

© 2022. Published by The Company of Biologists under the terms of the Creative Commons Attribution License (https://creativecommons.org/licenses/by/4.0/).

Holly R. Brooker, Michael A. Geeves, Daniel P. Mulvihill (2016) Analysis of biophysical and functional consequences of tropomyosin-fluorescent protein fusions. FEBS Letters Volume590, Issue18 September 2016 Pages 3111-3121.

Martin C, Schevzov G and Gunning P (2010) Alternatively spliced N-terminal exons in tropomyosin isoforms do not act as autonomous targeting signals. J Struct Biol 170, 286-293.

Urbancikova M and Hitchcock-DeGregori SE (1994) Requirement of amino-terminal modification for striated muscle alpha-tropomyosin function. J Biol Chem 269, 24310-24315.

Hitchcock-DeGregori SE and Heald RW (1987) Altered actin and troponin binding of amino-terminal variants of chicken striated muscle alpha-tropomyosin expressed in Escherichia coli. J Biol Chem 262, 9730-9735.

3/ I am not sure I understand the authors' conclusion about the locations of Tpm1 and Tpm2 in cerevisiae. Could the authors clearly state whether they can conclude on a different localization pattern of these two tropomyosins, or whether this apparent difference is rather due to imaging limitations?

Response: With the experiments performed in this work, it appears that they localize similarly, but that Tpm2 signal is weaker than that of Tpm1. We mention this in the discussion with the following narrative. We are now making Tpm2 with the photostable stay gold protein to assess this question more convincingly.

We note that although mNG-40L-Tpm1 and mNG-40L-Tpm2 show similar distribution, the intensity of Tpm2 in various structures is weaker and more prone to photobleaching compared to Tpm1. In the future, we intend to make fusions of Tpm2 with the far superior and photostable Stay Gold protein. Such an analysis will clarify if Tpm1 and Tpm2 differ in their distribution and / or dynamics.

4/ Supp Figure 4E gives the impression that not all stress fibers are equally decorated by TPM2. Is this true or a misleading impression?

Response: We believe the striated pattern of Tropomyosin TPM2 localization in RPE cells is a true reflection in this cell type. We are now investigating detailed localization of TPM2 (and other TPMs) in RPE and other mammalian cell types to further examine the localization properties of mammalian tropomyosin isoforms.

<u>Second decision letter</u>

MS ID#: JOCES/2022/260288

MS TITLE: mNG-tagged fusion proteins and nanobodies to visualize tropomyosins in yeast and mammalian cells

AUTHORS: Tomoyuki Hatano, Tzer Chyn Lim, Ingrid Billault-Chaumartin, Anubhav Dhar, Ying Gu, Teresa Massam-Wu, William Scott, Sushmitha Adishesha, Bernardo Chapa-y-Lazo, Luke Springall, Lavanya Sivashanmugam, Masanori Mishima, Sophie G Martin, Snezhana Oliferenko, Saravanan Palani, and Mohan K Balasubramanian
ARTICLE TYPE: Tools and Resources

I am happy to tell you that your manuscript has been accepted for publication in Journal of Cell Science, pending standard ethics checks.

© 2022. Published by The Company of Biologists under the terms of the Creative Commons Attribution License (https://creativecommons.org/licenses/by/4.0/).
